# Multiagent-Based Data Presentation Mechanism for Multifaceted Analysis in Network Management Tasks

**DOI:** 10.3390/s22228841

**Published:** 2022-11-15

**Authors:** Kazuto Sasai, Ryota Fukutani, Gen Kitagata, Tetsuo Kinoshita

**Affiliations:** 1 Graduate School of Science and Engineering, Ibaraki University, Hitachi 316-8511, Japan; 2 Research and Education Faculty, Humanities and Social Science Cluster, Education Unit, Kochi University, Kochi 780-8072, Japan; 3 Department of English Language and Culture, Faculty of Humanities, Morioka University, Takizawa 020-0605, Japan; 4 Research Institute of Electrical Communication, Tohoku University, Sendai 980-8577, Japan

**Keywords:** data analytics, network management, multiagent system, serendipity, information recommendation, data presentation

## Abstract

Although network management tasks are highly automated using big data and artificial intelligence technologies, when an unforeseen cybersecurity problem or fault scenario occurs, administrators sometimes directly analyze system data to make a heuristic decision. However, a wide variety of information is required to address complex cybersecurity risks, whereas current systems are focused on narrowing the candidates of information. In this study, we propose a multiagent-based data presentation mechanism (MADPM) that consists of agents operating data-processing tools that store and analyze network data. Agents in MADPM interact with other agents to form data-processing sequences. In this process, we design not only the composition of the sequence according to requirements, but also a mechanism to expand it to enable multifaceted analysis that supports heuristic reasoning. We tested five case studies in the prototype system implemented in an experimental network. The results indicated that the multifaceted presentation of data can support administrators more than the selected single-faceted optimal presentation. The final outcome of our proposed approach is the provision of a multifaceted and cross-system data presentation for heuristic inference in network management tasks.

## 1. Introduction

As information and communications technology (ICT) has become indispensable in our lives, addressing increasingly complex cybersecurity risks has become a major issue. The sophisticated automation of network equipment has allowed non-experts to become administrators. However, when unexpected events occur, data must be analyzed and heuristic inferences must be made, which is a heavy burden for non-experts and a potential risk to network infrastructure. Therefore, there is a need for more advanced automation and support technology for network administrators. Autonomic network management [1,2,3] was proposed for the unmanned operation of network infrastructure. The idea is based on autonomic computing [4,5], which defines the components of network systems as autonomously acting and interacting as in biological organization. In recent years, AI researchers have attempted to develop the concept of the autonomic functions of network management by introducing machine learning [6,7,8]. However, because advanced machine learning has complex internal structures and representations, once unexpected behavior occurs, human administrators cannot know what is occurring in systems [9]. “Explainable AI” is anticipated to be able to provide a solution to the problem [10,11,12]; however, relevant technologies have not been established yet. Thus, complete automation using AI technology is not available now.

As an alternative approach to the unpredictability of the real world, researchers have introduced cooperation between humans and AI. Human-in-the-loop (HITL) [13,14] is a concept representing highly synthetic systems involving interaction between humans and machines. Humans are expected to provide feedback and introduce creativity to the precise calculations of machines. For large-scale information systems, the concept of human-agent collectives (HAC) [15] has been proposed as a diverse cooperating community of humans and agents. The requirements of HACs assert that agents should have functions of *accountability* to be trusted by humans. Accountability and creativity provide the communication scheme for HITL and HAC concepts. For example, accountability is supported by providing information from internal systems and humans contribute a novel perspective while understanding the information, for example, annotations for machine learning [16,17]. Therefore, how to provide and present information from the agent (machine) side is a key challenge in HITL and HAC systems.

Recently, data management and analytics have increased their importance in the context of big data and the IoT. Big data has certain problems, not only with the size of databases, but also with the heterogeneity that reflects the multiplicity of formats, storage types, locations, and sensors [18]. Therefore, techniques for appropriately handling various types of data have been investigated [19,20]. The third problem for big data analytics is how to visualize big data [21,22]. Because of the limited space available for visualization, such as by use of laptops and smartphones, developers should select what data to store and how to display it appropriately. Therefore, for more advanced data analysis, it is necessary to fully consider the functions for resolving data diversity and selecting appropriate visualization elements.

For network management tasks, researchers have focused on the collection of security data [23,24] and IoT applications [25,26]. In parallel, several software tools have been used to compose data-processing flows or visualization formats. For instance, Elasticsearch (ELS) [27] and InfluxDB (IDB) [28] provide a flexible database and processing modules for heterogeneous data, for example, logs and time series. Fluentd [29], Logstash [30], and Kibana [31] provide real-time stream processing and visualization for server logs. General network management systems, such as Zabbix (ZBX) [32], provide device and service statuses and notify users of these statuses. We refer to the range of data-processing software applications for network management as *data-processing tools (DPTs)*. An adequately organized set of DPTs can strongly support network and server administrators. However, because composition and selection require professional knowledge about advanced ICT trends, some difficulties remain for non-professional, casual, and private engineers. Therefore, in this study, we propose an architecture to manage DPTs as autonomous agents that cooperate and self-organize.

Multiagent-based solutions have been applied to reduce complexity using cooperation among agents [33,34]. We have developed a multiagent-based network management system based on the concept of an active information resource (AIR), which is an information resource that autonomously interacts with other AIRs [35]. We extend the system to include data-analytic functions [36]. The idea for this study originated from the concept of AIR. However, existing systems focus on responding precisely to requests and lack extensions to generate ad hoc reasoning. Therefore, it is difficult to take effective measures against unpredictable and unknown scenarios. This is common for other general human-centered [37,38] and context-centered approaches [39,40].

Satisfying requests that concern creativity is called *serendipity* [41,42]. Technically, theory refers to a balance or trade-off between *accuracy* and *creativity* (expansion), similar to the trade-off in the exploration of optimal solutions [43]. A recommendation with expansion may correspond to the awareness of a hidden underlying relationship in the mind of a human. As mentioned above, the management problem in complex ICT systems requires human intelligence heuristics. We adopt the idea of serendipity to address the capability for multifaceted analysis for network management and apply and integrate it into a multiagent-based approach for network data management. Consequently, in this study, we propose a multiagent-based data-presentation mechanism (MADPM) to integrate heterogeneous DPTs and demonstrate beneficial processing results.

The main contributions of this study are as follows:

- We connect data analytics in network management and the idea of serendipity, which is the notion of an innovative encounter, to solve the complexity of recent cybersecurity problems.
- We propose a multiagent-based mechanism to provide a multifaceted data presentation (MADPM).
- We define the organization process considering accuracy and expansion for user demand. Additionally, we include automated composition of the data presentation process in the organization process.
- We present a design process to encapsulate services and systems as agents (data-processing agents (DPAs)), including data related to network management.
- We conducted several case studies and demonstrated that the multifaceted information provided by the prototype system enabled us to make inferences from new perspectives.
- Finally, we discuss the effectiveness and feasibility of the data and information recommendation, including uncertainty represented by the probabilistic process.

This paper is an extended version of the conference paper [44] and provides details of the algorithms and case studies.

## 2. Materials and Methods

### 2.1. Multiagent-Based Data Presentation Mechanism (MADPM)

**Figure 1:**
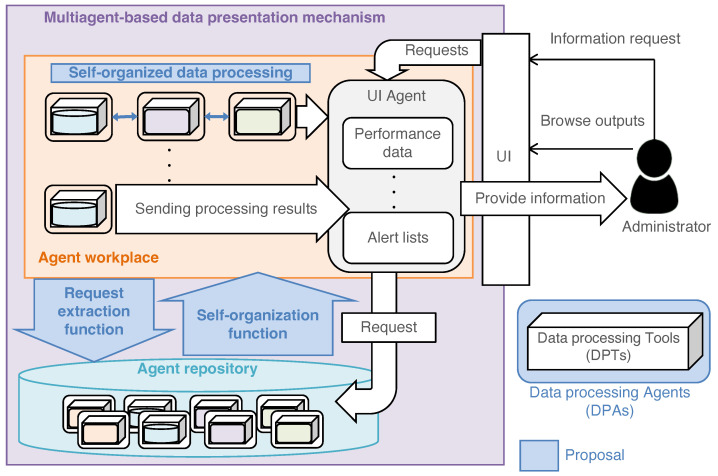
Schematic diagram of the multiagent-based data presentation mechanism (MADPM). The proposed MAPDM mainly consists of the encapsulation of data-processing tools (DPTs) and autonomous composition of multiagent organizations.

In this study, we use *presentation* because the mechanism composes the data-processing flow and recommends the result related to user demand. Figure 1 shows a schematic diagram of the MADPM. First, we create DPAs that include DPTs with the knowledge and functions for handling DPTs. Self-organization means that the interaction between agents establishes the data-processing flow required for providing information according to the request input by users. The design scheme follows the distributed agent system-based hybrid architecture (DASH) framework [45]. The self-organization process proceeds in the agent repository, which is a unique feature of the framework, and the generated organization is activated in the workplace. Finally, data-processing starts.

As shown in Figure 1, we use a user interface (UI) agent (UIA) to serve and manage a UI, which receives the request and returns the results from the active DPAs in the workplace. Thus, the notions of *accuracy* and *expansion* are expressed in the organization processes of *request-matching* and *request expansion*, respectively.

#### 2.1.1. Request Matching

As shown in Figure 1, when a user inputs a request for data-processing into the UI, the UIA sends a broadcast message of the request to the agent repository. The request is expressed as a triplet:

(1) $$R:=\{r_{i}|r_{i}=<target_{i},data_{i},processing_{i}>;i=1,2,3,\cdots ,n\},$$

where $target_{i}$ denotes the name of the target device, $data_{i}$ denotes the name of the data (fields), and $processing_{i}$ denotes the name of the requested type of processing. Using appropriate natural language processing methods, the triple is extracted from request *r* described by a natural language expression. For example, when a request to know the current central processing unit (CPU) usage, “Show CPU usage of the mail server”, occurs, the entities of the triple $r_{i}$ are extracted, such as $target_{i}=“\mathrm{Mail}\mathrm{server}”$, $data_{i}=“\mathrm{CPU}\mathrm{usage}”$, and $processing_{i}=“\mathrm{provide}”$. The entities are used to evoke the functions of DPAs.

In the DASH framework, the program that defines the decision-making of the agent’s behavior is called “agent knowledge”. In this paper, the response to the received request is determined by agent knowledge. We design two possible types of agent knowledge: agent knowledge about data DK and agent knowledge about the processing procedure PK. DK describes what data are stored or measured in the DPA:

(2) $$DK:=\{dk_{i}|dk_{i}=<source_{i},variable_{i},description_{i}>;i=1,2,\cdots ,n\},$$

where source is the name of the data source, variable is the name of the measured variable observed on the source device, and description is the text that explains the data. By contrast, PK indicates agent knowledge about the processing method for the data:

(3) $$PK:=\{pk_{i}|pk_{i}=<processing_{i},keyword_{i},probability_{i}>;i=1,2,\cdots ,n\},$$

where processing is the name of the possible processing method, keyword is the keyword that triggers the execution of the data processing function, which corresponds to the activation of the DPA, and probability is the probability that the DPA is activated.

A DPA checks the correspondence between the incoming request $r_{i}$ and its own knowledge $dk_{i}$ and $pk_{i}$, and decides to start working. We introduce a probabilistic decision model to express flexibility in the organizing process. Note that the notion of decision flexibility is not new and is the basis of *fuzzyness* [46,47]. Our probabilistic decision model is defined using the activation probability $ap_{i}$ defined as

(4) $$ap_{i}=\left\{\begin{array}{cc} 1 & \left(target_{i}\subseteq source_{i}\right)\\ 1-\frac{\alpha (n_{i}^{rd}-n_{i}^{rkd})}{n_{i}^{rd}} & (otherwise), \end{array}\right.$$

where $n_{i}^{rd}$ is the number of nouns in $data_{i}$ of request $r_{i}$, and $n_{i}^{rkd}$ is the number of nouns included in $description_{i}$ of $dk_{i}$ and included in $data_{i}$ of $r_{i}$. The DPA is always activated if the target device $target_{i}$ matches the data source $source_{i}$. The term $n_{i}^{rd}-m_{i}^{rkd}/n_{i}^{rd}$ represents the matching rate between a request and the knowledge of a DPA. α∈[0.0,1.0] is an activation coefficient that can be set directly in the knowledge description. If α=0, then $ap_{i}=1$ indicates that the agent is always activated for any request. For example, when $data_{i}=“\mathrm{CPU}\mathrm{usage}”$ and $description_{i}=“\mathrm{CPU}\mathrm{utilization}\mathrm{rate}\mathrm{in}\mathrm{user}\mathrm{space}.”$, “CPU usage” can be divided into the nouns “CPU” and “usage,” then $n_{i}^{rd}=2$ and “CPU” is also included in $description_{i}$; therefore, $n_{i}^{rkd}=1$. Finally, if α=0.5 and $ap_{i}=0.75$, then the agent will be activated by 75%. Because the prototypical implementation in this study is for Japanese, we use a morphological analysis tool to extract the words from a request. The coefficient may be replaced with an algorithm to change the value temporally. This remains a topic for future work.

If the DPA does not have $dk_{i}$ but has $pk_{i}$, this means that the DPA does not store data but can be part of a data-processing flow; then the DPA only responds to requests $r_{i}$ for processing. For example, if processing in $pk_{i}$ is matched to that in $r_{i}$, the DPA is activated by the probability parameter in $pk_{i}$. At least one candidate is determined for the processing request, and then the process is completed.

#### 2.1.2. Request Expansion

**Figure 2:**
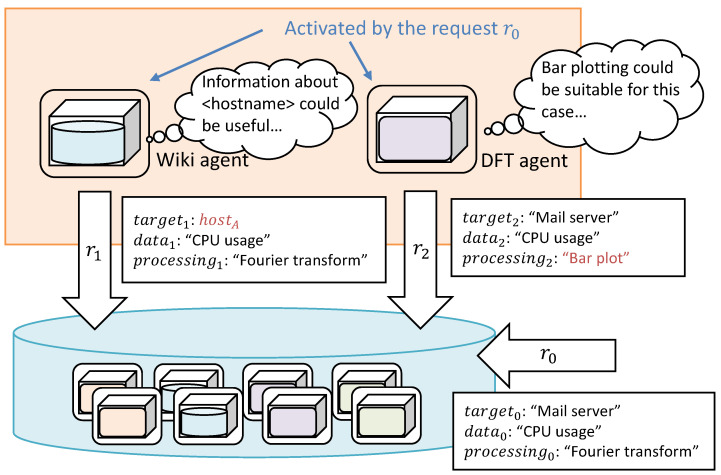
Example of request expansion. The activated DPAs can generate secondary requests, partially replaced by knowledge of the semantic relation. The mechanism provides a bottom-up expansion of demands in the request.

Mining hidden potential information is the core idea of serendipity [41]. We introduce request expansion to apply the idea in the self-organization process. The MADPM permits DPAs to generate a modified request in chains. Figure 2 shows an example of request expansion. Now, the initial request $r_{0}$ is received as

(5) $$r_{0}=<“\mathrm{Mail}\mathrm{server}”,“\mathrm{CPU}\mathrm{usage}”,“\mathrm{Fourier}\mathrm{transform}”>,$$

and then the two agents are activated. A possible example is that an abstract word, “Mail server”, can be concretized to the hostname $“host_{A}”$ on which mail server applications are installed. The DPA of the wiki (wiki agent), which has the management information shared by administrators, generates a secondary request:

(6) $$r_{1}=<“host_{A}”,“\mathrm{CPU}\mathrm{usage}”,“\mathrm{Fourier}\mathrm{transform}”>,$$

and sends the agent repository. Another possible example is that the activated DPA requires subsequent processes, such as plotting. As shown in Figure 2, a DPA of a discrete Fourier transformation (DFT) generates a secondary request $r_{2}$ as

(7) $$r_{2}=<“\mathrm{Mail}\mathrm{server}”,“\mathrm{CPU}\mathrm{usage}”,“\mathrm{Bar}\mathrm{plot}”>.$$

Notably, not only the hidden demands of the information, but also the data processing flows, can be composed using the expansion type in Equation (7).

We have defined the matching and expansion processes in this section; the combination provides helpful information for heuristic problem solving in network management. Note that the mechanism should be designed considering the possibility of convergence.

### 2.2. Data Processing Agent (DPA)

**Figure 3:**
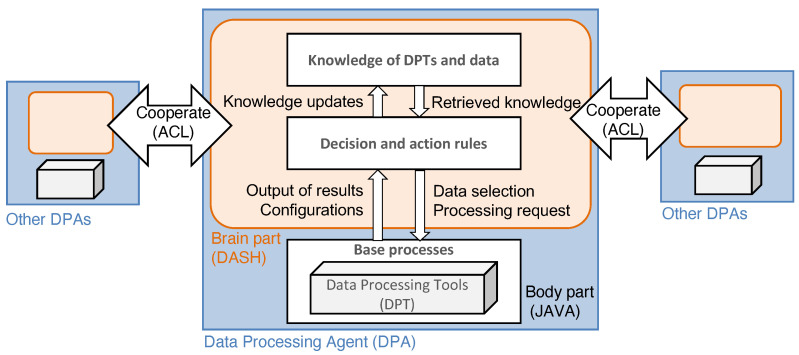
Conceptual diagram of a DPA. The blue part indicates the entire body of the DPA; the orange part indicates the brain component, which consists of knowledge and the decision rules; and the white part corresponds to the original DPT.

In this section, we define the structure of a DPA. Figure 3 indicates a schematic diagram of a DPA. DPAs are the agents that use DPTs, for example, ELS [27] and ZBX [32]. The ADIPS/DASH [45] framework divides an agent into the two design components of brain and body parts. The brain component describes agent knowledge and actions used to interact with the other agents. For example, when receiving a message from other agents or measuring environmental change, an agent decides what action should be taken for the recognized scenario using installed knowledge. According to the framework, the rule-based inference model describes the knowledge of an ADIPS/DASH agent. The body component is called the “base process”, which is a process that the wrapper for the DPT program uses to provide the actions triggered in the brain component. We describe the details of each component in the following sections.

#### 2.2.1. Knowledge and Action Rules

**Figure 4:**
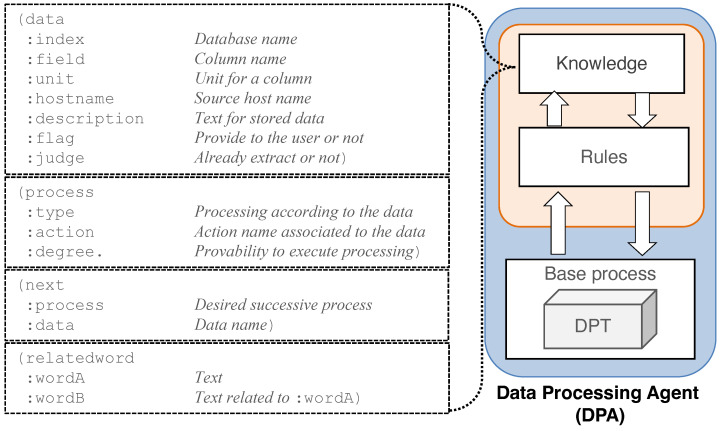
List of data-processing knowledge for the DPA. A dashed square indicates each knowledge element. Each knowledge element is defined as a separate agent knowledge.

Figure 4 shows the list of entities used to construct agent knowledge for data processing. Agent knowledge is written in object-attribute-value format and divided into four types: “data”, “process”, “next” and “related_word”. “data” is agent knowledge used to obtain data from the particular DPTs that store logs or performance data. “process” stores the possible functions of processing and calculating in the DPT. “next” contains the names or keywords of the processing method that the DPA recommends to perform the next process. For example, if the DPA acquires the time series of network traffic data and wants to plot it on a line graph, we can write the value of attribute “process” as “Line plot”. If the DPA has additional information to provide to the user, it can be defined in the knowledge of “relatedword”. Using this knowledge, for instance, if “Web server” is related to “$host_{A}$”, which is a particular hostname, we can write the values of the attributes “wordA” and “wordB”.

#### 2.2.2. Base Process

**Figure 5:**
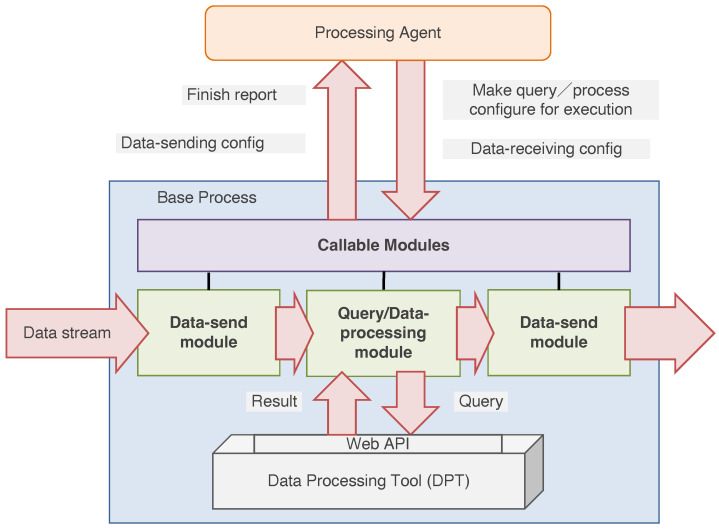
Structure diagram of a base process in a DPA. The blue part represents the base process. The green boxes are modules that transfer data and process them via DPT functions. The purple box represents methods callable from the agent (brain) part.

The DPA can control the existing or original DPTs using encapsulation. The encapsulated program is called the base process in the ADIPS/DASH framework. Figure 5 shows the structure of a base process. Encapsulation consists of four modules: the module that the agent calls directly, the data-receiving module, the query module, and the data-sending module. The “data-sending module” directly exchanges data with other agents by establishing the data stream. Note that the agent can configure and control the data stream at the level of agent interaction, which refers to the message exchange using knowledge defined in the previous section. Then, the received data are transferred to the “data-processing module” to process and execute the query in the DPT. The results of DPT-processing or the query are sent to other agents via the data-sending module.

**Figure 6:**
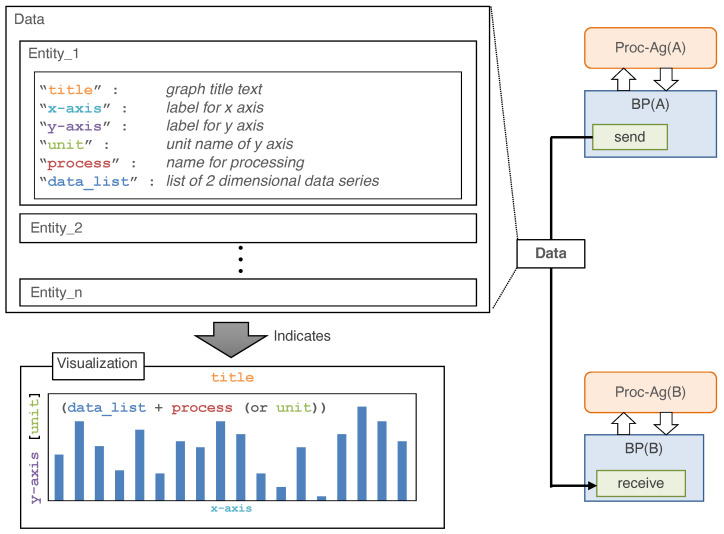
Structure of data exchanged between the base processes. Data are delivered as a piece of variables with metadata in JSON format.

Figure 6 depicts the data exchanged via the data-sending module. Data are delivered as components of variables with metadata in JSON format. The metadata include “title”, “*x*-axis”, “*y*-axis”, “unit”, “process” and “data_list”. In this study, because the prototypical implementation defines terminal processing as visualization, the information required to draw a graph is delivered as part of the metadata. “process” defines the type of graph. If “process” is not specified, the graph type is inferred from the description of “unit”. A code example of the base process is included in the Supplementary Materials.

#### 2.2.3. Lifecycle

**Figure 7:**
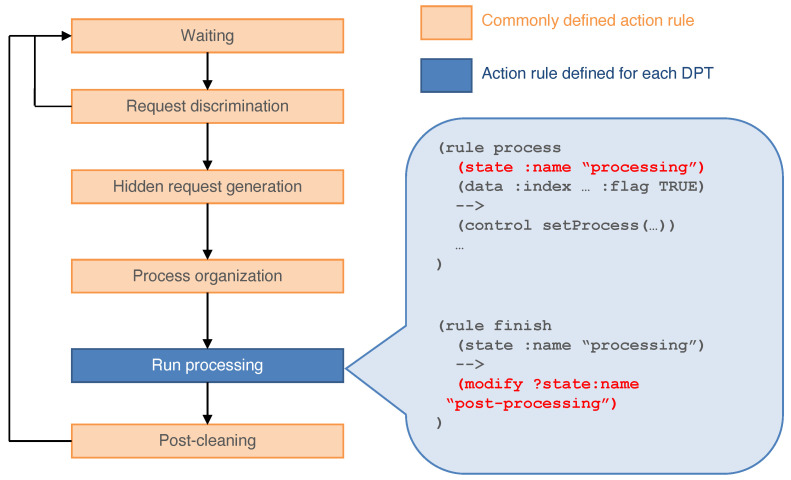
Action flow diagram for the DPA. We developed the common parts (templates) of the steps in orange. When encapsulating another DPT, the blue part must be implemented.

Figure 7 is a diagram of the lifecycle of a DPA. The DPA in the agent repository waits for request messages from other agents. When it receives a message, the DPA checks whether the request is acceptable by verifying it with the knowledge. If the request is not acceptable, the message is dropped and the state returns to the waiting state. If the requested process can run, the DPA is instantiated in the workplace. The DPAs in the repository support the processing flow. Because the process is recursive, a processing flow that answers the request is organized. Then, the DPA establishes a direct connection between the base processes of the before and after processes of the data processing sequence. After the processing flow is organized, the state becomes “processing” and data processing is run. When the agent has completed the processing task, the state of the agent changes to “post-processing”. Then, the DPA returns to the repository. The sample codes of DPAs are shown in Appendix A and Appendix B.

### 2.3. Prototype Implementation

In this section, we implement a prototypical system to demonstrate the MADPM practically and evaluate its advantages. First, we consider a broad set of monitoring tools for a small- or medium-scale network as follows:

- *Log collection tool* collects logs on the servers, such as mail servers, web servers, and firewalls, and displays statistics and time series.
- *Performance management tool* collects performance data, such as CPU usage, memory usage, and the amount of traffic on network interfaces from the servers and networking devices, and provides statistics and visualization.
- *Service management tool* provides the function of service management, which includes monitoring, testing, and providing alerts on the service status.
- *Knowledge tool* stores knowledge for management tasks and provides functions to share among employees.

#### 2.3.1. Experimental Environment

**Figure 8:**
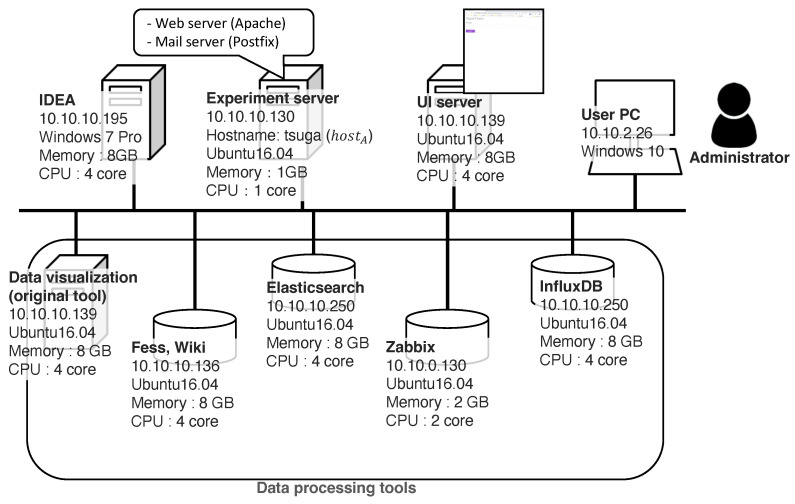
Experimental environment for the prototype system. We deployed four physical servers and five DPTs on the servers. The virtual fault scenarios occurred on the “experimental server”, and the experimenter (administrator) demonstrated the case studies using a “User PC” via the “UI server”.

Figure 8 shows a diagram of the experimental environment. We conducted experiments on our laboratory network with the experimental servers, which included the five DPTs listed in Table 1. We executed all the implemented agents on IDEA [45], which is a development and runtime tool for ADIPS/DASH agents. The experimental server included a web server and a mail server process to test the case study described later in this paper. We conducted experiments on a test PC connected to the same subnet. The ELS and IDB tools were used on different ports on the same server for convenience.

#### 2.3.2. Implemented Agents

**Figure 9:**
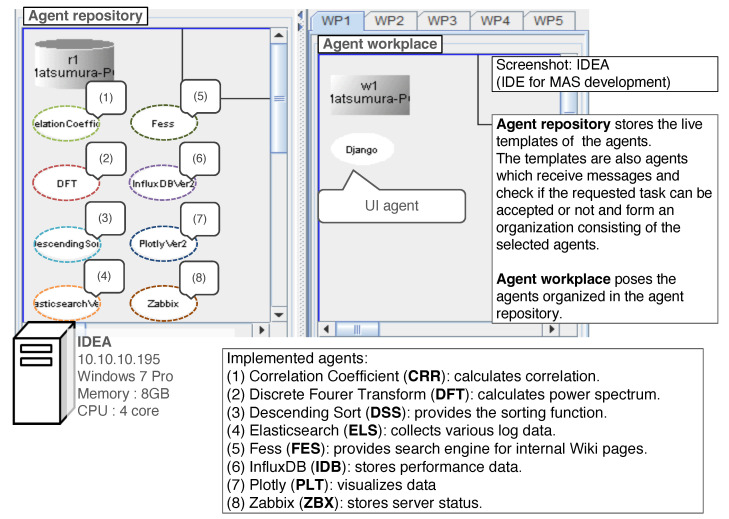
Screenshot of the initial state of the prototype system with descriptions. We implemented eight DPAs and a user interface (UI) agent. The MADPM works among the agents.

Figure 9 shows a screenshot of the runtime environment window of IDEA when the system started (initial state). According to the ADIPS/DASH framework regulations, the runtime environment included the agent workplaces and repository. The agent repository stored agents that were inactive but capable of exchanging messages. We implemented eight DPAs that appeared in the agent repository. We designed each agent according to the MADPM. We provide a brief description of the agents below.

##### Correlation Coefficient (CRR) Agent

The CRR agent provides the Pearson correlation coefficient for the received time series using the preliminary stored anomaly time series. Simultaneously, the CRR agent sends the anomalous time series to other agents, such as the data visualization agent. When CRR is in the repository, if the received request $r_{i}$ includes “Correlation analysis”, then CRR is activated by 100%, if $r_{i}$ includes “Analysis”, then CRR is activated by 80%, otherwise, CRR is activated by 10%. To compare the present time series with the visualized anomaly time series, CRR rewrites $process_{i}$-entity in $r_{i}$ as “Line plot” and sends it to the repository.

##### Discrete Fourier Transform (DFT) Agent

DFT is an original agent that provides the power spectrum calculated from the received time series data. The agent is activated by 1000% if the name of process in request $r_{i}$ is “Fourier transform”, 80% if process is “Analysis”, and 10% otherwise. DFT replaces process with “Bar plot” to visualize the power spectrum.

##### Descending Sort (DSS) Agent

DSS is also an original agent that sorts the received time series in order of the average value. This agent aims to present important data first. DSS is activated by 100% if process is “Descending sort”, 80% if process is “Analysis”, and 10% otherwise. DSS also rewrites process as “Plot”.

##### Elasticsearch (ELS) Agent

ELS is an agent that encapsulates ELS [27] software for collecting logs from servers. Through the functions on ELS that use query operations, the agent provides a function to retrieve log data, retrieve the count per minute, and construct time series. We define three types of log data on the experiment server with the hostname “tsuga” as the knowledge of the agent part of ELS. Then, ELS is activated by 100% if the target device name target in $r_{i}$ is “$host_{A}$”. The knowledge of data can be improved to extend the function to retrieve monitoring hostnames from the database. As an additional action, if process is “provide”, ELS rewrites process as “Bar plot”.

##### FESS (FES) Agent

FES encapsulates the search engine FES [48] for local sites. FES can retrieve multiple sites. The FES agent provides search results for the search keyword from local wiki sites or other services in the local network. The agent can respond by 100% to every request. If target is “Web server” or “Core switch,” the agent rewrites target as “$host_{A}$” or the IP address of the core switch, respectively. We require that the action rule is constructed from knowledge in the wiki and aim to extract it automatically in the future.

##### InfluxDB (IDB) Agent

IDB encapsulates the OSS database engine IDB [28]. The agent provides time series by retrieving and calculating the CPU usage, memory usage, and amount of traffic according to the received request $r_{i}$. In this study, we implement five entities and ten entities for the experiment server and core switch, respectively. When the agent returns the data, we set the tic of the time series to one minute. IDB is activated by 100% if the target device target is “$host_{A}$” or the IP address of the core switch. If process in $r_{i}$ is “Provide”, then the agent rewrites it as “Line plot”.

##### Plotly (PLT) Agent

PLT is an agent that encapsulates the data visualization tool PLT [49]. When the agent receives time series data, it returns an HTML file with the corresponding graph. For example, PLT is activated by 100% if process is one of “Line plot”, “Bar plot” or “Plot” and 10% otherwise.

##### Zabbix (ZBX) Agent

ZBX encapsulates the popular NMS, ZBX [32], which monitors and analyzes hosts and switches in the target networks. The agent provides the result from the retrieved health status of the target hosts. The ZBX agent is always activated for any request.

#### 2.3.3. User Interface Agent (UIA)

**Figure 10:**
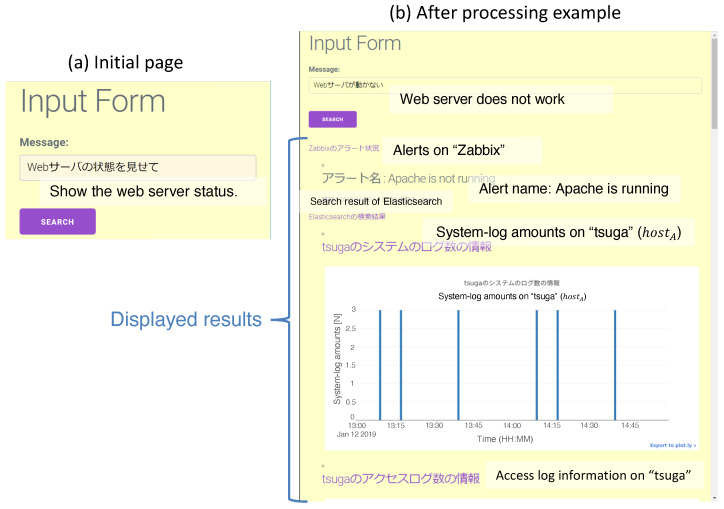
Screenshot of the UI server: (**a**) An initial state. We can input the request for the system in natural language and data processing starts when the user pushes the search button. (**b**) Example result after processing is complete. The results are ordered and displayed below the input form.

We implemented the UIA using the Django framework in the Python language. Figure 10 shows screenshots of the UI. The UI contains the request form and the search button for launching analytics. After the MADPM process, the results are displayed below the input form.

## 3. Results

### 3.1. Experiments with the Prototype System

We conducted experiments using the prototype system to evaluate the MADPM. In [44], we tested two cases and demonstrated the advantage of the MADPM. In this paper, we present five more case studies for the prototype system. In each case, all the phenomena are caused manually so that the actual operation of the laboratory is not affected.

### 3.2. Case 1: Trouble on the Web Server

Human error is a major reason for system failures. In this case, we demonstrate misconfiguration on the web server. The specific procedure for the experiment is as follows:

1. halt the process of “apache2.service” on the “monitored server” (Figure 8)
2. access the UI server and input the test request as “Web server is not running”.
3. check the returned list of information.

**Figure 11:**
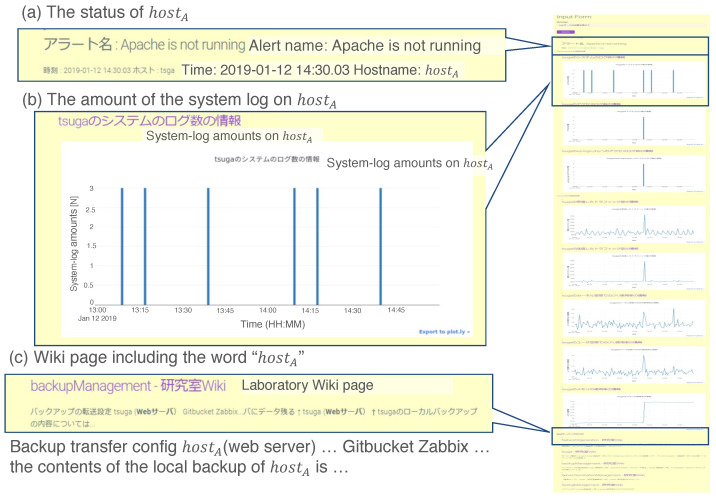
Example result for the prototype system in Case 1. We assert three remarkable results: (**a**) status of $host_{A}$, (**b**) amount of the system log on $host_{A}$, and (**c**) wiki page including the word “$host_{A}$”.

Figure 11 shows the output of the prototypical system. Figure 11a shows an alert on the ZBX server. The alert indicates “Apache is not running”, with the hostname of the “monitored server” (displayed hostname is $host_{A}$). Figure 11b shows a time series of the system logs; below are the access logs and performance data. The shape of the graph in Figure 11b looks periodic, which implies that the system worked normally before it halted. Figure 11c shows corresponding information on the knowledge management system (wiki) of the experimental (lab.) network. From the output of (c), the administrator can know complementary information about failure detection.

**Figure 12:**
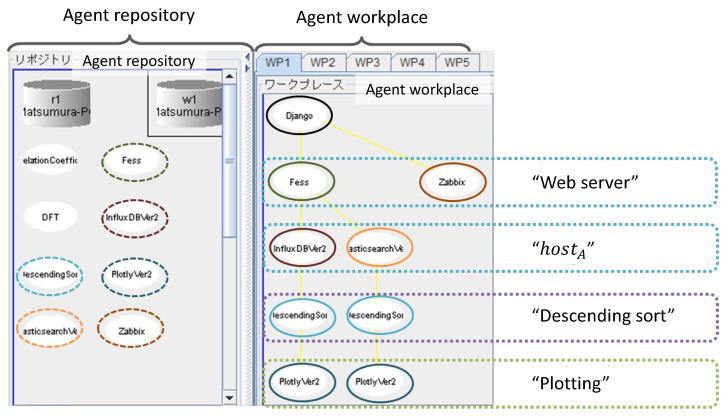
Screenshot of the agent runtime environment (IDEA) when the result of Case 1 was displayed. In this case, six agents were activated from the agent repository and eight active agents organized the three data-processing streams.

Figure 12 shows a screenshot of the agent repository and workplaces when the output of Figure 11 was displayed. The actual self-organization process executed for the request is presented as follows:

1. The UIA generates the request as
  (8) $$r_{1}=<“\mathrm{Web}\mathrm{server}”,“\mathrm{NULL}”,“\mathrm{Analysis}”>$$
  and sends it to the agent repository.
2. The FES and ZBX agents appear in the workplace according to the entity “Web server” in $r_{1}$.
3. The FES agent generates another request $r_{2}$ as
  (9) $$r_{2}=<“host_{A}”,“\mathrm{NULL}”,“\mathrm{Analysis}”>$$
  and sends it to the agent repository.
4. The IDB and ELS agents respond to “$host_{A}$” in $r_{2}$ and come into the workplace. The two agents resend $r_{2}$ to the agent repository.
5. The two DSS agents corresponding to the IDB and ELS agents are instantiated with “Descending sort” in $r_{2}$.
6. From the relation in the knowledge of the DSS agent, a new request $r_{3}$ is created as
  (10) $$r_{3}=<“host_{A}”,“\mathrm{NULL}”,“\mathrm{Plot}”>$$
  and sent to the agent repository.
7. The PLT agent is instantiated with “Plot” in $r_{3}$.
8. The processing flow is automatically organized through the IDB, DSS, and PLT agents.

The result confirms the self-organized mechanism to verify that complex user demand works expectedly.

### 3.3. Case 2: Amount of Traffic on the Network Switch

Case 2 presents the reaction to the request for providing performance information over time on network devices, for example, time series of the amount of traffic on Ethernet ports. Performance information over time is a popular monitoring entity for an enterprise-scale system and dedicated workers monitor this 24 hours a day, 7 days a week. However, because it is difficult to cover this type of work for small networks, such as laboratory networks, an administrator checks traffic on demand or when it is troubling. In this case, we test the following process:

1. Input the phrase “Show the amount of traffic on the core switch in descending order”.
2. Check the output information.

**Figure 13:**
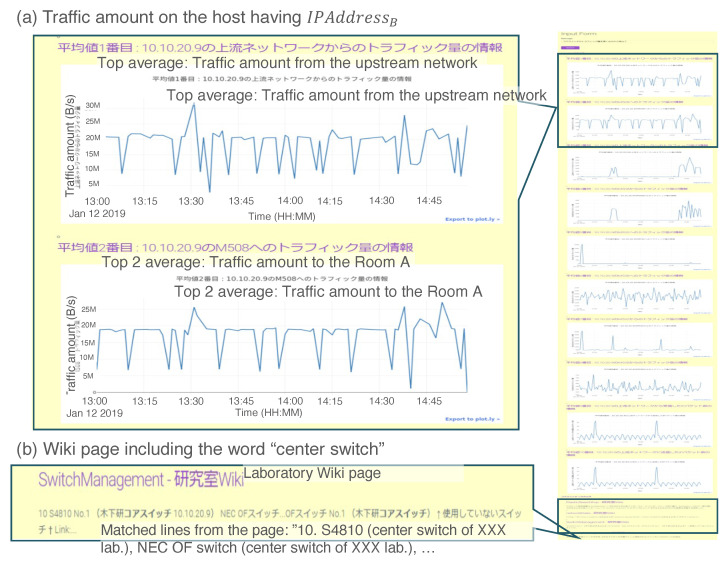
Example result for the prototype system in Case 2. We assert two remarkable results: (**a**) the amount of traffic on the host with $IPaddress_{B}$ and (**b**) the wiki page includes the word “core switch”.

Figure 13 shows a screenshot of the output for the above test input. The top of the UI (Figure 13a) displays the upstream (WAN) traffic and that into Room A. There is also knowledge of a device (core switch) with the same IP address. From the input of “the core switch”, the administrator can know not only the amount of traffic on the switch, but also management information to understand the consistency of the IP address, hostname, and role. Because administrators are often not fixed in small networks, inferences that are effective for problem-solving can be made by displaying not only visualized time-series data, but also shared knowledge about the target server within the laboratory.

The flow for the organization and providing data for the input request is as follows:

1. UIA generates the request triple as
  (11) $$r_{1}=<“\mathrm{Core}\mathrm{switch}”,“\mathrm{Amount}\mathrm{of}\mathrm{traffic}”,“\mathrm{Descending}\mathrm{sort}”>$$
  and sends $r_{1}$ to the agent repository as the broadcasting message, which is sent to all agents in the repository.
2. The FES agent is activated by reacting to the keyword “Core switch” and instantiated in the workplace.
3. Using the stored knowledge, the FES agent newly generates another request:
  (12) $$r_{2}=<“IPaddress_{B}”,“\mathrm{Amount}\mathrm{of}\mathrm{amount}”,“\mathrm{Descending}\mathrm{sort}”>.$$
4. The IDB and ELS agents respond to “<IPaddress B>” in $r_{2}$ and come into the workplace.
5. The DSS agent is instantiated with “Descending sort” in $r_{2}$.
6. From the relation in the knowledge of the DSS agent, a new request $r_{3}$ is created as
  (13) $$r_{3}=<“IPaddress_{B}”,“\mathrm{Amount}\mathrm{of}\mathrm{amount}”,“\mathrm{Plot}”>$$
  and sent to the agent repository.
7. The PLT agent is instantiated with “Plot” in $r_{3}$.
8. The processing flow is automatically organized through the IDB, DSS and Plotting agents.

### 3.4. Case 3: Denial of Service (DoS) Attack

A DoS attack stops the service by sending huge transactions to the server or service network. In this scenario, the administrator should quickly investigate the network and take sufficient action to stop the damage. In this case, we use the Apache JMeter [50] to obtain the load on the target server. The test procedure consists of the following steps:

1. Generate five transactions from a terminal to “$host_{A}$” per second.
2. After a while, generate 60 transactions from another terminal to “$host_{A}$” per second.
3. Confirm that the website on “$host_{A}$” is down.
4. Access the UI server and input “$host_{A}$ is down”.
5. Check the output of the UI server.

**Figure 14:**
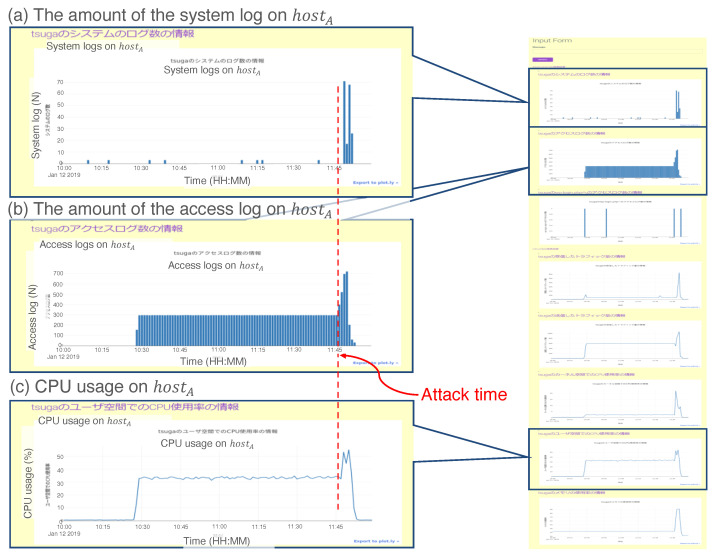
Example result for the prototype system in Case 3. We assert three remarkable results: (**a**) the amount of the system log on $host_{A}$, (**b**) the amount of the access log on $host_{A}$, and (**c**) central processing unit (CPU) usage on $host_{A}$.

Figure 14 shows a screenshot of the output displayed on the UI server. For visibility, we drew a red dashed line to indicate the attack time of Step 2 above. Figure 14a shows the number of system logs on $host_{A}$, which rapidly increased at the attack time. The logs provided notification of the data collection failure from the data collector agent on $host_{A}$. Additionally, the amount of access logs (Figure 14b) and CPU usage (Figure 14c) of $host_{A}$ increased simultaneously. From the three results in (a)–(c), the administrator could infer the existence of a DoS attack at that time.

In this case, the prototypical system reacted to the test input “$host_{A}$ is down.” and organized the agents as follows:

1. The UIA generates the request as
  (14) $$r_{1}=<“host_{A}”,“\mathrm{NULL}”,“\mathrm{analysis}”>$$
  and sends it to the repository.
2. The IDB and ELS agents appear in the workplace according to the entity “$host_{A}$” in $r_{1}$.
3. The two agents resend $r_{1}$ to the repository.
4. The DSS agent is instantiated in the workplace according to “Analysis” in $r_{1}$.
5. The DSS agent generates another request $r_{2}$ as
  (15) $$r_{2}=<“host_{A}”,“\mathrm{NULL}”,“\mathrm{Plotting}”>$$
  and sends it to the repository.
6. The PLT agent is instantiated in the workplace by “Plot” of $r_{2}$.
7. The processing flow of the data is automatically constructed.

### 3.5. Case 4: Brute Force Attack

A brute force attack is a cyberattack that challenges various patterns of passwords for logging into the target host. In this case study, we virtually generate a brute force attack on “$host_{A}$” and check the output of the prototypical system. The experimental procedure is as follows:

1. Access “$hoat_{A}$” from a terminal five times per second.
2. Generate a virtual brute force attack on “$host_{A}$” ten times per minute.
3. After continuing Step 2 for 2 hours, input “Analyze the logs on $host_{A}$”.
4. Analyze the output from the prototypical system.

**Figure 15:**
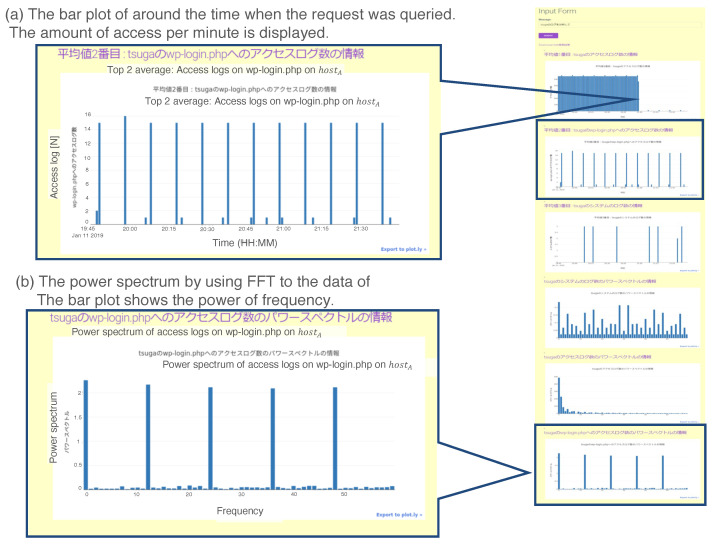
Example result for the prototype system in Case 4. Two results are as follows: (**a**) The bar plot of the time when the request was queried. The amount of access per minute is displayed. (**b**) The power spectrum using DFT on the queried data. The bar plot shows the power of the frequency.

Figure 15 shows a screenshot of the output of the above experiment. Figure 15a indicates there were periodic login challenges to the page “/wp-login.php” on “$host_{A}$”. Figure 15b shows the Fourier analysis result for the data in Figure 15a. The graph also indicates the periodic activity of login challenges; the red circled spike represents n=12, which equals 10 minutes. By integrating the two results, the administrator could suspect a brute force attack on $host_{A}$.

The processing flow in the prototypical system when it receives the request “Analyze the logs on $host_{A}$.” is as follows:

1. The UIA generates request $r_{1}$ from the input text as
  (16) $$r_{1}=<“host_{A}”,“\mathrm{Logs}”,“\mathrm{Analysis}”>$$
  and sends it to the agent repository.
2. The ELS agent is activated by reacting to the keyword “$host_{A}$” and instantiated in the workplace.
3. Using the stored knowledge, the ELS agent resends $r_{1}$ to the agent repository.
4. The DFA and DSS agents are activated by reacting to the keyword “Analysis” and instantiated in the workplace.
5. Using the stored knowledge, the DFA agent newly generates another request $r_{2}$ as
  (17) $$r_{2}=<“host_{A}”,“\mathrm{Logs}”,“\mathrm{Bar}\mathrm{plot}”>$$
  and sends it to the DPAs in the agent repository.
6. Using the stored knowledge, the DSS agent newly generates another request $r_{3}$ as
  (18) $$r_{3}=<“host_{A}”,“\mathrm{Logs}”,“\mathrm{Plotting}”>$$
  and sends it to the DPAs in the agent repository.
7. The PLT agent is instantiated with “BarPlotting” in $r_{2}$ and “Plot” in $r_{3}$.
8. The processing flow is automatically organized through the instantiated agents.

### 3.6. Case 5: Data Correlation for Anomaly Detection

As a particular use case for the prototypical system, correlation analysis among heterogeneous data on network equipment provides a good demonstration. For instance, a method using the correlation between two different measures of network traffic was proposed for network monitoring [11].

In this case study, we conduct an experimental analysis of the inter-temporal correlation between CPU usage data. The experimental flow is as follows:

1. Generate access to $host_{A}$ five times per second.
2. Input “Analyze the CPU usage on $host_{A}$.” to the UI server.
3. Check the output from the prototypical system.

**Figure 16:**
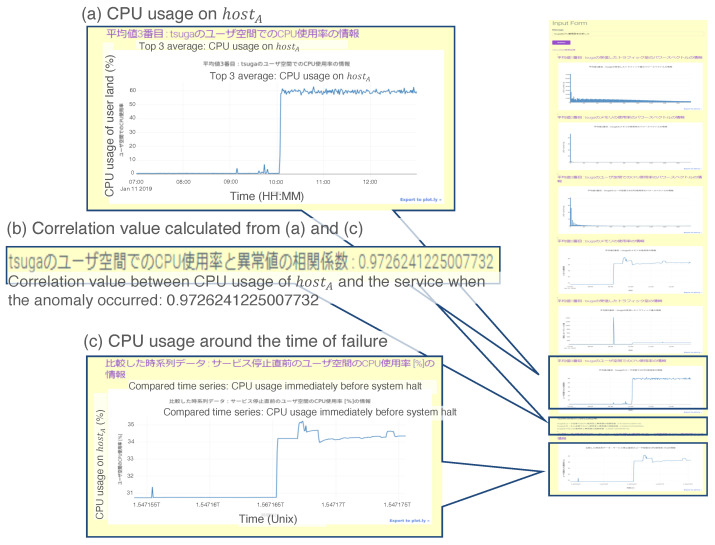
Example result for the prototype system in Case 5. We assert three remarkable results: (**a**) CPU usage on $host_{A}$, (**b**) correlation value calculated from (**a**,**c**), and (**c**) CPU usage around the time of the anomaly occurrence.

Figure 16 shows a screenshot of the output for the experiment described above. Figure 16a,c indicate the time series when the request was input and when an anomaly occurred in the past, respectively. Furthermore, Figure 16b shows the correlation value between the data in (a) and (c). Because the correlation value was 0.97 in the case study, the administrator could infer that something wrong occurred on $host_{A}$.

The processing flow in the prototypical system when it receives the request “Analyze the CPU usage on *Host_A_*.” is as follows:

1. The UIA generates request $r_{1}$ from the input text as
  (19) $$r_{1}=<“host_{A}”,“\mathrm{CPU}\mathrm{usage}”,“\mathrm{Analysis}”>$$
  and sends it to the agent repository.
2. The ELS and IDB agents are activated by reacting to the keyword “*Host_A_*” and instantiated in the workplace.
3. Using the stored knowledge, the ELS and IDB agents resend $r_{1}$ to the agent repository.
4. The CRR and DSS agents are activated by reacting to the keyword “Analysis” and instantiated in the workplace.
5. Using the stored knowledge, the CRR agent newly generates another request $r_{2}$ as
  (20) $$r_{2}=<“host_{A}”,“\mathrm{CPU}\mathrm{usage}”,“\mathrm{Line}\mathrm{plot}”>$$
  and sends it to the DPAs in the agent repository.
6. Using the stored knowledge, the DSS agent newly generates another request $r_{3}$ as
  (21) $$r_{3}=<“host_{A}”,“\mathrm{CPU}\mathrm{usage}”,“\mathrm{Plotting}”>$$
  and sends it to the DPAs in the agent repository.
7. The PLT agent is instantiated with “Line plot” in $r_{2}$ and “Plot” in $r_{3}$.
8. The processing flow is automatically organized through the instantiated agents.

## 4. Discussion

### 4.1. Effectivity and Heterogeneity

**Figure 17:**
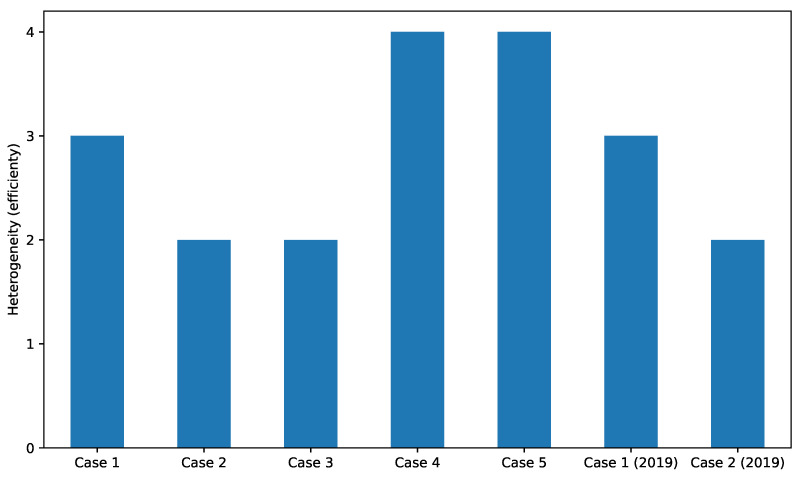
Comparison of heterogeneity, which is counted using the types of information provided by the prototype system. If the administrators (experimenters) collect the same types of information, they have the burden of accessing and operating the systems.

To evaluate the MADPM, we compared the burden on human administrators by counting how many databases are used to create results. We chose this number because the manual process takes the same steps if the human uses different systems to acquire the number of results. Figure 17 shows the counted heterogeneity and number of databases for the presented information. We define the heterogeneity of the data presentation as if the results had been collected from different data analytics systems. For example, the output of Case 1 was collected from three types of DPTs, that is, ZBX, ELS, and FES, as shown in Figure 11. If an administrator performs the same task manually, this generates three times the burden of the prototype system because the administrator has to use at least three systems to collect the same data. The result indicates that the proposed self-organization scheme can efficiently collect and present heterogeneous data using the multiagent cooperation mechanism.

Table 2 shows a comparison of the functional coverage of data presentation between the traditional approach and MADPM. The traditional approach uses tools for respective types of network data. For example, ELS is normally used to collect logs because of its advanced search capability. The remarkable sets of outputs shown in Figure 11 and Figure 13, Figure 14, Figure 15 and Figure 16 can be provided for each tool individually. However, cross-sectional findings are only provided by the proposed MADPM. As discussed in the previous section, functionality is realized by the cooperation and autonomy of DPAs.

### 4.2. Performance and Scalability

Because our MADPM includes the request expansion process, *combinatorial explosion* can occur. For example, in Figure 6 in the original conference paper [44], we measured the processing time by increasing the quantity of data stored in the system. As a result, there was a difference between DPTs: in the case of the FES agent, even if the number of items on the wiki site used as the search source was increased from 20 to 100, there was no significant difference in processing time. This result was caused by the performance of the FES software. By contrast, the processing time increased linearly according to the number of graphs generated by the PLT agent. Considering practical uses, DPAs are designed to exhibit the capability of DPTs. Additionally, we have not investigated the scalability of increasing the number of agents because of the complexity. We have not provided a solution in this paper for processing performance. In many research fields, load-balancing methods for data processing have been studied, for example, edge computing [34], and can be applied to MAPDM.

### 4.3. Limitations

**Figure 18:**
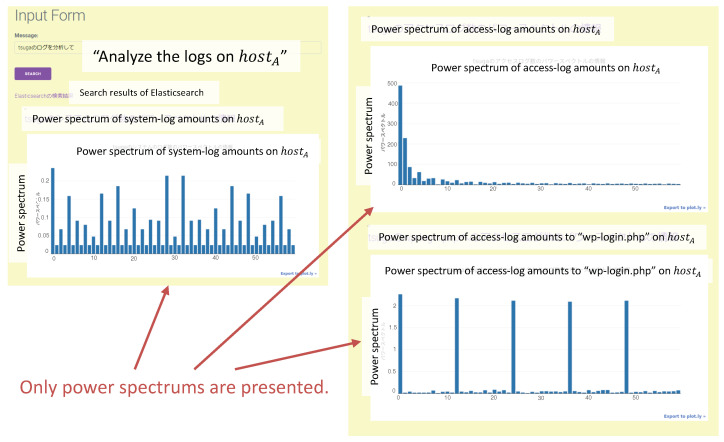
Example of the undesirable output. In this case, the request “Analyze the logs of $host_{A}$.” was input. The system output only the power spectrums of the logs, but it was not a high-priority analysis for the target data.

In the prototype system, the output variety depends on the probability coefficient α. In Case 4, for the request “Analyze the logs of $host_{A}$”, the outputs of (a) and (b) in Figure 15 were presented with a probability of 72%. By contrast, if the request “Analyze the data of $host_{A}$” was input, the same outputs were provided with a probability of 36%. The description in $dk_{i}$ caused the difference because it was written as “The number of access logs for the login page.” in the log acquisition (ESS) agent. The probability $ap_{i}$ was 1 if data in $r_{i}$ was “Logs”; however, $ap_{i}$ was 0.5 if data was “data”, where α=0.5. Figure 18 shows the output of the unfavorable result. In this case, the request “Analyze the logs of $host_{A}$“ was input into the system. However, the output was only power spectrums, which are not advisable in this scenario. The current prototype system design and implementation cannot respond sufficiently to a variation in notation. This should be improved in a future version.

### 4.4. Implications

Although advanced network infrastructure management has become possible, cyberattacks and system failures remain unresolved for a long period. Current network management systems quickly provide information on serious scenarios that occur in systems and rapid communication systems also allow users to report any problems they notice quickly. Conventionally, a failure recovery process is defined such that, after an event is detected, the cause is diagnosed and necessary recovery work is performed. However, in unpredictable circumstances, such as a sudden increase in network usage caused by the effect of COVID-19, problems may occur in unexpected places, and recovery may take longer than expected. In such cases, the proposed system can cause the administrator to notice the cause of the problem, which is usually difficult to determine by displaying information from different perspectives in response to a request from the administrator.

We can also address the issue of the education of non-professional administrators. Newly appointed administrators do not yet know the details of the network. The MADPM, which proposes a method to collect related information using a flexible free word search, is an effective mechanism for such administrators because it can simultaneously provide domain knowledge in addition to current data analysis. Although cross-system cooperation must handle a huge amount of relational information, the proposed approach solves this problem by distributing it well and selecting it stochastically using a multiagent mechanism.

## 5. Conclusions

We proposed a multiagent-based mechanism to present data analytics for network management tasks. We introduced accuracy and expansion into request-matching for the agent organization process by considering creative heuristics in exploratory analysis. We designed agents to encapsulate the DPTs that handle and process network management data. We implemented the prototype system in an experimental network to evaluate the proposed approach. We conducted five case studies on the experimental network using the prototype system. Through the case studies, multifaceted result presentations supported the exploration task for cause detection. Even though AI-based automation technologies are spreading widely, the demand for human creativity will remain while the cause of cybersecurity risks comes from humans. Our proposed approach can contribute to technologies empowering humanity in the use of practical systems. As discussed in Section 4, the limitations of flexibility and parameter-tuning remain to be addressed in future work. The final goal of our proposed approach is to achieve multifaceted and cross-system data presentation.

## Figures and Tables

**Table 1:** List of data collection tools deployed in the experimental network.

Name	Description
InfluxDB [28]	a performance management tool
Elasticsearch [27]	a log collection tool
Zabbix [32]	a service management tool
Fess [48] and Wiki	a knowledge tool (Fess provides the search engine for Wiki)
Data visualization	an original tool implemented by Python

**Table 2:** Comparison of function coverage for data presentation.

	Case 1			Case 2		Case 3			Case 4		Case 5		
Traditional tools	(a)	(b)	(c)	(a)	(b)	(a)	(b)	(c)	(a)	(b)	(a)	(b)	(c)
InfluxDB [28]		X		X				X			X		X
Elasticsearch [27]		X				X	X		X				
Zabbix [32]	X												
Fess [48] and Wiki			X		X								
MADPM	X	X	X	X	X	X	X	X	X	X	X	X	X

**Listing A1:**
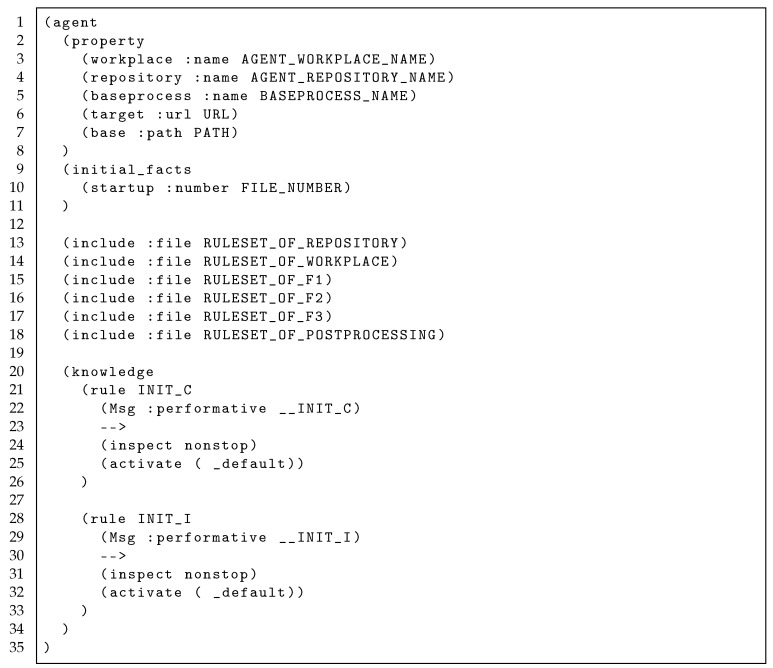
Template of agents.

**Listing A2:**
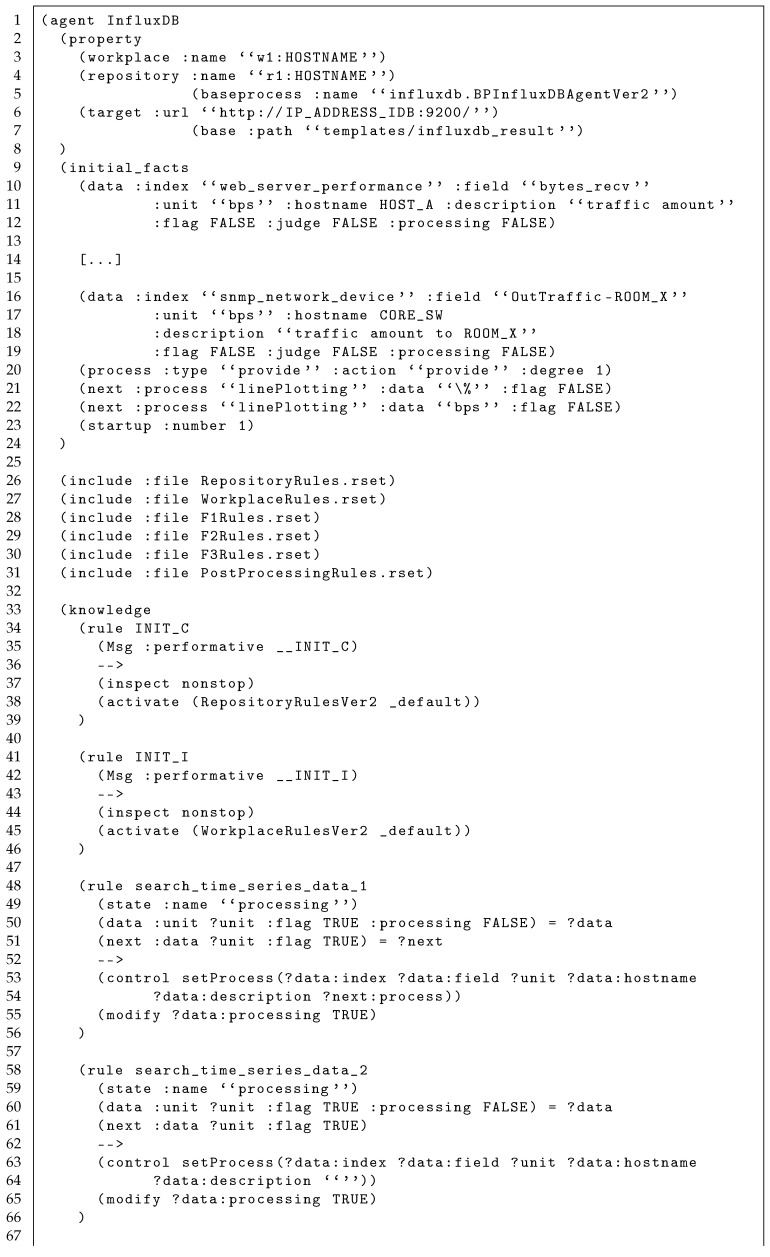

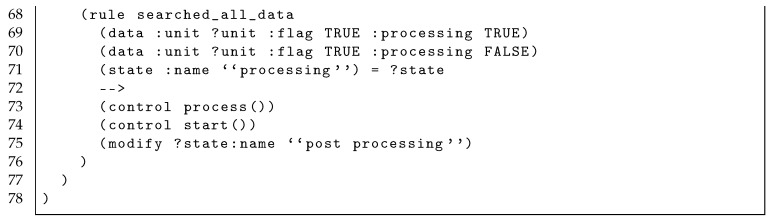
Sample of InfluxDB agent.

**Listing A3:**
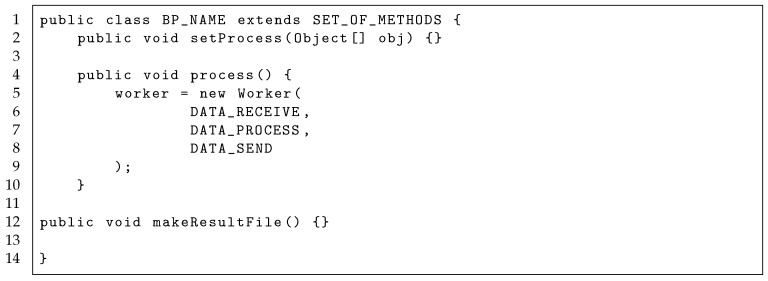
Template of base process.

**Listing A4:**
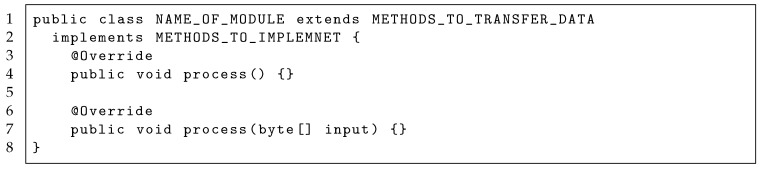
Template for data processing modules.

**Listing A5:**
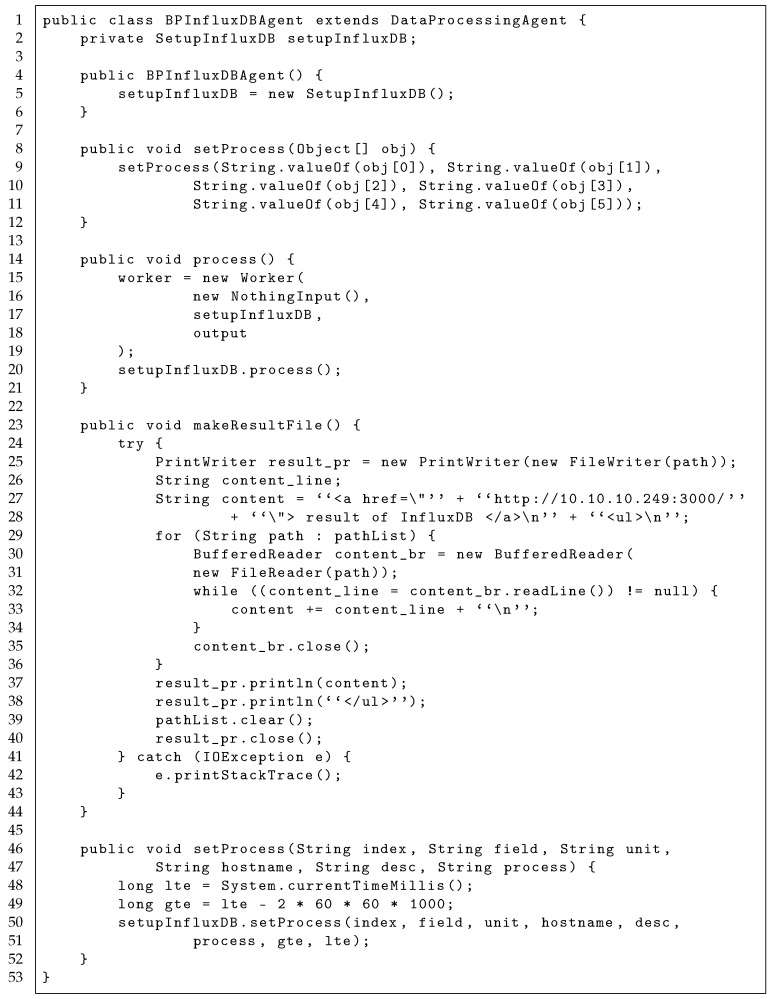
Sample of base process (InfluxDB agent).

**Listing A6:**
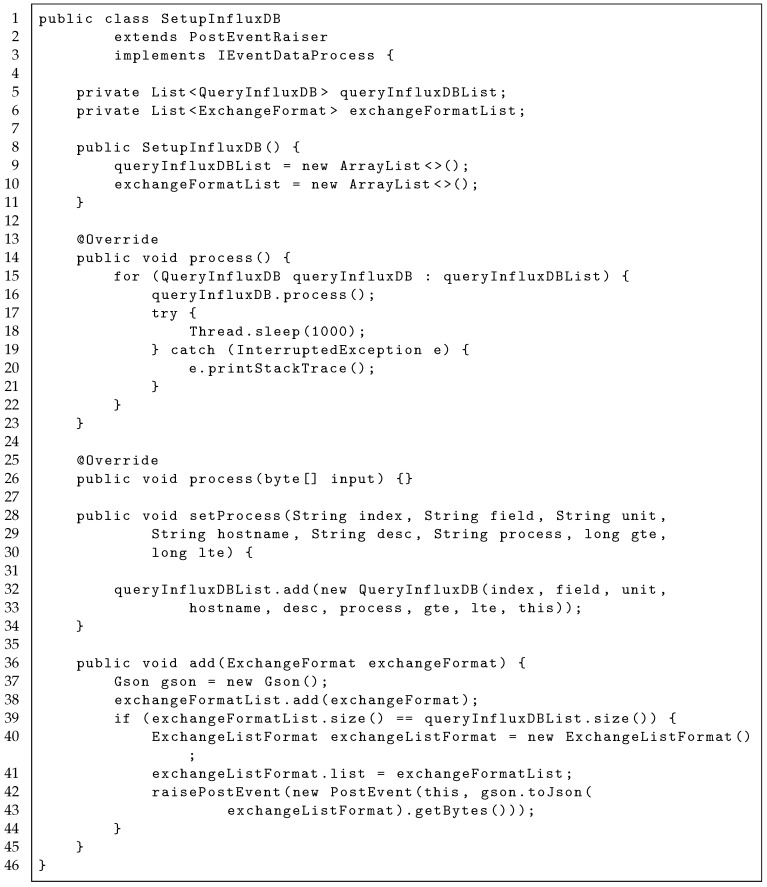
Sample of data process module of IDB agent.

## Data Availability

The data presented in this study are available in the article and supplementary material.

## Supplementary Materials

The following supporting information can be downloaded at: https://www.mdpi.com/article/10.3390/s22228841/s1, Listing S1: Rule set file of repository; Listing S2: Rule set for workplace; Listing S3: Rule set file of F1; Listing S4: Rule set file of F2; Listing S5: Rule set file of F3; Listing S6: Rule set file of post processing; Listing S7: Class file for base process; Listing S8: Data process module.

## Appendix A. Sample Code of DPA

The source code developed in this study is described according to the DASH framework [45] and built by the interactive design environment for agent system (IDEA) [51]. The readers can execute the codes using IDEA based on the appropriate settings for the user environment. According to the DASH framework, we should prepare two types of files, DASH (.dash) files and base processes written in Java language (.java).

### Appendix A.1. DASH File

Listing A1 presents a template that describes the common parts of the DPAs implemented in this paper. The template already contains the default agent workspace name, agent repository name (AGENT_REPOSITORY_NAME), file number (FILE_NUMBER), rule set to read (RULESET_OF_*), and two types of rules (INIT_I, INIT_C). The attribute values of knowledge on lines 5 to 7 of the code are values unique to the data-processing tool, so they should be described by the developer. The described rules are written so that, when the agent is placed in the repository, the repository ruleset is read, and, when the agent is placed in the workplace, the workplace ruleset is loaded.

### Appendix A.2. Example of IDB Agent

Listing A2 is an example code of implemented DPAs. In this case, the DPA is specially built for InfluxDB. Lines 13 to 18 include action rules common to DPAs. The rules for each file are described in the Supplementary Materials. This code is the data-processing agent that controls the data-processing tools (IDB) managing the performance data. The 6th to 8th lines of the code describe the name of the base process to be loaded, the URL of the data processing tool to be controlled and the path of the result output file, respectively. The 10th and 14th lines correspond to knowledge $dk_{i}$ and describe the amount of traffic received by HOST_A and the amount of traffic flowing to ROOM_X of the core switch (CORE_SW), respectively. The description on the 17th line describes the processing performed by this data-processing agent. In this example, when the processing $p_{i}$ to be performed on the data of request $r_{i}$ is “provide”, the agents that perform line graph drawing described in lines 21 and 22 are called. The rules described below the 47th line execute data processing. Lines 49, 59, and 71 are limited to cases where the knowledge of (state :name “processing”) is retained, and, on line 75, the knowledge is changed (modify) to (state :name “post-processing”).

## Appendix B. Sample Code of Base Process

### Appendix B.1. Templates of Base Process

Listing A3 is a template of the base process for DPTs. The base process inthe DASH framework should be written in Java language. We have to define the DATA_RECEIVE, DATA_PROCESS and DATA_SEND to provide callable functions and a communication interface for the other agents, as designed in Section 2.2.2. The first line inherits a class file that describes methods common to the base process. The class file is described in the Supplementary Materials. The setProcess method on the second line is a method that performs necessary settings for the data-processing module (DATA_PROCESS). The Worker class on the 5th line is a class for passing data between the receiving, processing and sending modules. The data-receiving module (DATA_RECEIVE) described in the 6th line is described as “input” when input from other agents is required and described as “new NothingInput()” when not required. The data-receiving module described in line 6 is described as “output” when outputting data to other agents and described as “new NothingOutput()” when not outputting data. The makeResultFile method on the 11th line is prepared as a method for integrating the processing results.

Listing A4 is a template for the data-processing module. The process method on the 4th line is a method for executing data processing according to control messages from the agent. The process method on line 7 is a method that is executed when data is received from the data receive module. The received data is “input” in the byte array of the argument.

### Appendix B.2. Example Codes of Base Process

Listing A5 and Listing A6 show example descriptions of the base process and data-processing module in the IDB agent, respectively. In this example, the class corresponding to the query creation/data-creation module is “SetupInfluxDB”. Since this module acquires time-series data from the database, “new NothingInput()” is written on the 15th line of Listing A3 and no code is written in the process method on the 23rd line of Listing A6. The process method on the 13th line executes the query necessary for data retrieval every second. The add method on line 32 receives the search results from “QueryInfluxDB” and, when all the issued queries are processed, the data is converted to a byte array and passed to the data send module.
