# Supplementary material for: Multiagent-Based Data Presentation Mechanism for Multifaceted Analysis in Network Management Tasks
Source: Sensors (Basel). 2022 Nov 15;22(22):8841. doi: 10.3390/s22228841 (PMC9697569; doi:10.3390/s22228841)
Supplement: Supplementary file 1 [file sensors-22-08841-s001.zip › sensors-1986322-supplementary.pdf]

Supplementary Materials: Rule Sets and Class Files

Kazuto Sasai, Ryota Fukutani, Gen Kitagata and Tetsuo Kinoshita

As part of the data availability, this document provides rulesets for the Java classes and distributed agent system-based hybrid architecture (DASH) framework [1] that are commonly used in the programs created in this research, and is distributed for the purpose of promoting reuse by readers.

1. Implementation of Rule Sets

The DASH agent framework provides OAV (Object-Attribute-Value) format for rule set description. The rule sets provide the common action rules for each agent.

1.1. Repositoty

Listing S1 is the rule set for data processing agents (DPAs) in the repository. The rules on the 12th and 37th lines are a group of rules executed by a data processing agent that converts the database into an agent. This rule activates the agent when it receives a message whose performative attribute is "`request notification`" and the device attribute of the search object in the request content matches the data it holds. The rule on line 62 is for the data processing agent that holds network monitoring information. When a message with the performative attribute of "`request notification`" is received, it is generated in the operating environment regardless of its contents. The rule on line 135 is a rule that fires when a message whose performative attribute is "`task notification`" is received. When this rule fires, the `judgeProcess` method commonly implemented in the base process is executed to determine whether the agent is activated. If the judgment result is "`TRUE`", the rule on the 162nd line is executed, and if it is "`FALSE`", the rule on the 189th line is executed. The rules on lines 202 and 213 are rules for deleting messages that do not correspond to the above.

Listing S1: Rule set file of Repository

```
1  (rule-set RepositoryRulesVer2
2
3    (property)
4    (initial_facts)
5
6    (rule load_BP
7      (baseprocess :name ?name)
8      -->
9      (loadBP ?name)
10   )
11
12   (rule receive_request_notification_to_DBAg_1
13     (Msg
14       :performative "request notification"
15       :from ?agName
16       :to _broadcast
17       :departure ?dep
18       :content (search :device ?dev :target ?tar :action ?action)
19     ) = ?msg
20     (data :hostname ?dev)
21     (process :type ?action)
22     (startup :number ?num) = ?startup
23     (workplace :name ?wp)
24     -->
25     (instantiate :into ?wp :facts (
26       (files :number ?num)
27       (notification :from ?agName :departure ?dep)
28       (search :device ?dev :target ?tar :action ?action :relevance 1.0)
29       (Members :manager ?agName :contractor ())
30       (process :type ?action :flag TRUE))
```

```
  )
  (bind ?number (+ ?startup:number 1))
  (modify ?startup:number ?number)
  (remove ?msg)
)

(rule receive_request_notification_to_DBAg_2
  (Msg
    :performative "request notification"
    :from ?agName
    :to _broadcast
    :departure ?dep
    :content (search :device ?dev :target ?tar :action ?action)
  ) = ?msg
  (data :hostname ?dev)
  (process :type ?action)
  (startup :number ?num) = ?startup
  (workplace :name ?wp)
  -->
  (instantiate :into ?wp :facts (
    (files :number ?num)
    (notification :from ?agName :departure ?dep)
    (search :device ?dev :target ?tar :action ?action :relevance 1.0)
    (Members :manager ?agName :contractor ())
    (process :type ?action :flag FALSE))
  )
  (bind ?number (+ ?startup:number 1))
  (modify ?startup:number ?number)
  (remove ?msg)
)

(rule receive_request_notification_to_DBAg_3
  (Msg
    :performative "request notification"
    :from ?agName
    :to _broadcast
    :departure ?dep
    :content (search :device ?dev :target ?tar :action ?action)
  ) = ?msg
  (accept :target ?dev)
  (data :hostname ?dev)
  (process :type ?action)
  (startup :number ?num) = ?startup
  (workplace :name ?wp)
  -->
  (instantiate :into ?wp :facts (
    (files :number ?num)
    (notification :from ?agName :departure ?dep)
    (search :device ?dev :target ?tar :action ?action :relevance 1.0)
    (Members :manager ?agName :contractor ())
    (process :type ?action :flag TRUE))
  )
  (bind ?number (+ ?startup:number 1))
  (modify ?startup:number ?number)
  (remove ?msg)
)

(rule receive_request_notification_to_DBAg_4
  (Msg
    :performative "request notification"
    :from ?agName
    :to _broadcast
    :departure ?dep
    :content (search :device ?dev :target ?tar :action ?action)
  ) = ?msg
  (accept :target ?dev)
  (data :hostname ?dev)
  (process :type ?action)
  (startup :number ?num) = ?startup
```

```
    (workplace :name ?wp)
    -->
    (instantiate :into ?wp :facts (
      (files :number ?num)
      (notification :from ?agName :departure ?dep)
      (search :device ?dev :target ?tar :action ?action :relevance 1.0)
      (Members :manager ?agName :contractor ())
      (process :type ?action :flag FALSE))
    )
    (bind ?number (+ ?startup:number 1))
    (modify ?startup:number ?number)
    (remove ?msg)
  )

  (rule receive_request_notification_to_InfoAg
    (Msg
      :performative "request notification"
      :to _broadcast
      :content (search :device ?dev :target ?tar :action ?action :from User)
    ) = ?msg
    (data :index NONE)
    (startup :number ?num) = ?startup
    (workplace :name ?wp)
    -->
    (instantiate :into ?wp :facts (
      (files :number ?startup:number)
      (notification :from ?msg:from :departure ?msg:departure)
      (search :device ?dev :target ?tar :action ?action :relevance 1.0)
      (Members :manager ?msg:from :contractor ()))
    )
    (bind ?number (+ ?startup:number 1))
    (modify ?startup:number ?number)
    (remove ?msg)
  )

  (rule receive_task_notification_to_ProcAg_1
    (Msg
      :performative "task notification"
      :content (search :action ?action)
    )
    (data)
    (process :type ?proc :action ?action :degree ?deg)
    //(process :type ?proc :action ?action :degree ?degree)
    (check)
    -->
    (make (check :flag TRUE))
    (control judgeProcess(?proc ?deg))
  )

  (rule receive_task_notification_to_ProcAg_2
    (Msg
      :performative "task notification"
      :content (search :action ?action)
    )
    (data)
    (process :type ?proc :action "ALL" :degree ?deg)
    (process :type ?proc :action ?action)
    (check)
    -->
    (make (check :flag TRUE))
    (control judgeProcess(?proc ?deg))
  )

  (rule check_task_TRUE
    (Msg
      :performative __Event
      :content (process :type ?type :flag TRUE)
    ) = ?msg1
    (Msg
```

```
      :performative "task notification"
      :content (search :device ?dev :target ?tar :action ?action)
    ) = ?msg2
    (startup) = ?startup
    (workplace :name ?wp)
    (check) = ?check
    -->
    (instantiate :into ?wp :facts (
      (files :number ?startup:number)
      (notification :from ?msg2:from :departure ?msg2:departure)
      (search :device ?dev :target ?tar :action ?action :relevance 1.0)
      (Members :manager ?msg2:from :contractor ())
      (process :type ?type :flag TRUE))
    )
    (bind ?number (+ ?startup:number 1))
    (modify ?startup:number ?number)
    (remove ?msg1)
    (remove ?msg2)
    (remove ?check)
  )

  (rule check_task_FALSE
    (Msg
      :performative __Event
      :content (process :type ?type :flag FALSE)
    ) = ?msg1
    (Msg :performative "task notification") = ?msg2
    (check) = ?check
    -->
    (remove ?msg1)
    (remove ?msg2)
    (remove ?check)
  )

  (rule ignore_irrelevant_request
    (Msg
      :performative "request notification"
      :content ((search :relevance ?rel) ?noti)
    ) = ?msg
    (relevance :border ?border)
    (> ?border ?rel)
    -->
    (remove ?msg)
  )

  (rule ignore_request_notification_1
    (Msg
      :performative "request notification"
      :content (search :device ?word)
    ) = ?msg
    (data)
    (data :hostname ?word)
    -->
    (remove ?msg)
  )

  (rule ignore_request_notification_2
    (Msg
      :performative "request notification"
      :content (search :device ?word)
    ) = ?msg
    (data)
    -->
    (remove ?msg)
  )

)
```

1.2. Workplace

Listing S2 is the rule set for the DPAs activated to the workplace. In this rule set, in addition to calling the base process, the rule sets for F1, F2, and F3 are switched.

Listing S2: Rule set for workplace

```
(rule-set WorkplaceRulesVer2

  (property)
  (initial_facts)

  (rule load_BP
    (baseprocess :name ?name)
    (target :url ?url)
    (files :number ?num) = ?files
    (base :path ?path)
    -->
    (loadBP ?name)
    (control setURL(?url))
    (control makePathName(?path ?num))
    (make (state :name "F1"))
    (remove ?files)
    (activate (F1Rules WorkplaceRulesVer2))
  )

  (rule receive_file_path_name
    (Msg
      :performative __Event
      :content (file :path ?path)
    ) = ?msg
    (Status :name ?myName)
    -->
    (make (file :path ?path :by ?myName))
    (remove ?msg)
  )

  (rule receive_output_info
    (Msg
      :performative __Event
      :content (output :type ?type)
    ) = ?msg
    -->
    (make ?type)
    (remove ?msg)
  )

  (rule activate_f2_rules
    (state :name "F2") = ?state
    -->
    (modify ?state:name "F2_1")
    (activate (F2Rules WorkplaceRulesVer2))
  )

  (rule activate_f3_rules
    (state :name "F3")
    -->
    (activate (F3Rules WorkplaceRulesVer2))
  )

  (rule activate_default_rules
    (state :name "processing")
    -->
    (activate (WorkplaceRulesVer2 _default))
  )

  (rule activate_post_processing_rules
    (state :name "post processing")
    -->
```

```
    (activate (PostProcessingRules WorkplaceRulesVer2))
  )
)
```

1.3. F1: Request matching

Listing S3 is the rule set of F1 which corresponds to the request matching property defined in Section 2.1.1 of the main manuscript. In this ruleset, it reports to the request issuer that it has been generated (lines 12 and 21), and from lines 37 to 77, each time-series data it holds is sent to the administrator. It is determined whether to provide to If there is more than one data to provide, he moves on to F2, and if there is none, it disappears from the workplace (line 120).

Listing S3: Rule set file of F1

```
1  (rule-set F1Rules
2
3    (property)
4    (initial_facts)
5
6    (rule send_request_acceptance
7      (notification :from ?agName :departure ?dep)
8      (data)
9      (Status :name ?myName)
10     (state :name "F1") = ?state
11     -->
12     (send
13       :performative "request acceptance"
14       :to ?agName
15       :arrival ?dep
16       :content (accepted :by ?myName)
17     )
18     (modify ?state:name "F1-check")
19   )
20
21   (rule send_task_acceptance
22     (notification :from ?agName :departure ?dep)
23     (data)
24     (process :flag TRUE)
25     (Status :name ?myName)
26     (state :name "F1") = ?state
27     -->
28     (send
29       :performative "task acceptance"
30       :to ?agName
31       :arrival ?dep
32       :content ()
33     )
34     (modify ?state:name "F2")
35   )
36
37   (rule match_attribute_hostname_and_description
38     (state :name "F1-check")
39     (search :device ?dev :target ?tar)
40     (data :description ?tar :hostname ?dev :flag FALSE :judge FALSE) = ?data
41     -->
42     (modify ?data:flag TRUE)
43     (modify ?data:judge TRUE)
44   )
45
46   (rule match_attribute_hostname_and_field
47     (state :name "F1-check")
48     (search :device ?dev :target ?tar)
49     (data :field ?tar :hostname ?dev :flag FALSE :judge FALSE) = ?data
50     -->
51     (modify ?data:flag TRUE)
52     (modify ?data:judge TRUE)
```

```
)

(rule match_attribute_hostname
  (state :name "F1-check") = ?state
  (search :device ?dev :target ?tar1)
  (data :description ?tar2 :hostname ?dev :flag FALSE :judge FALSE) = ?data
  (!= ?tar1 ?tar2)
  -->
  (control judge(?tar1 ?tar2))
  (modify ?data:judge JUDGING)
  (modify ?state:name "F1-judge")
)

(rule match_accept_target
  (state :name "F1-check") = ?state
  (accept :target ?dev1)
  (search :device ?dev1 :target ?tar1)
  (data :description ?tar2 :hostname ?dev2 :flag FALSE :judge FALSE) = ?
      data
  (!= ?tar1 ?tar2)
  (!= ?dev1 ?dev2)
  -->
  (control judge(?tar1 ?tar2))
  (modify ?data:judge JUDGING)
  (modify ?state:name "F1-judge")
)

(rule check_all_data_1
  (state :name "F1-check") = ?state
  (search :device ?dev)
  (data :hostname ?dev :flag TRUE) = ?data
  (data :hostname ?dev :flag FALSE :judge FALSE)
  -->
  (modify ?state:name "F2")
)

(rule check_all_data_2
  (state :name "F1-check") = ?state
  (search :device ?dev)
  (accept :target ?dev)
  (data :flag TRUE) = ?data
  (data :flag FALSE :judge FALSE)
  -->
  (modify ?state:name "F2")
)

(rule find_NONE_data
  (state :name "F1-check") = ?state
  (search)
  (data :index NONE)
  -->
  (modify ?state:name "F2")
)

(rule do_not_search
  (state :name "F1-check")
  (search :device ?dev)
  (data :hostname ?dev :flag FALSE :judge TRUE) = ?data
  (data :hostname ?dev :flag FALSE :judge FALSE)
  (notification :from ?agName :departure ?dep)
  (Status :name ?myName)
  -->
  (send
    :performative "processing failure"
    :to ?agName
    :arrival ?dep
    :content (failed :by ?myName)
  )
  (terminate :at now)
```

```
  )

  (rule receive_judgement_result
    (state :name "F1-judge") = ?state
    (data :flag FALSE :judge JUDGING) = ?data
    (Msg :performative __Event :content (?cont)) = ?msg
    -->
    (modify ?data:flag ?cont)
    (modify ?data:judge TRUE)
    (modify ?state:name "F1-check")
    (remove ?msg)
  )
)
```

1.4. F2: Secondary Requst Generation

ListingS4 is the rule set for F2 of secondary request generation is a part of request expansion property mentioned in Section 2.1.2 in the main text. The 6th or 74th line is the rule for calling the next process in the processing sequence of the secondary request generation function. When this rule fires, it sends a message whose performative attribute is “`task notification`” to the repository and waits for 10 seconds. Then, when the data processing agent wants to provide additional information to the user, the rule on line 117 is executed. In this rule, if there is a `relatedword` object, it rewrites the information of the target device of the request, sends a message whose performative attribute is “`request notification`” in the repository, and waits for 10 seconds. After that, we move to the rule that executes the function of F3.

Listing S4: Rule set file of F2

```
1  (rule-set F2Rules
2
3    (property)
4    (initial_facts)
5
6    (rule send_task_notification_1
7      (data :unit ?unit :flag TRUE) = ?data
8      (next :process ?proc :data ?unit :flag FALSE) = ?next
9      (next :process ?proc :flag TRUE)
10     (search) = ?search
11     (process :flag TRUE)
12     (repository :name ?name)
13     (state :name "F2_1") = ?state
14     -->
15     (modify ?search:action ?proc)
16     (send
17       :performative "task notification"
18       :to _broadcast
19       :arrival ?name
20       :content ?search
21     )
22     (modify ?state:name "F2_1-wait")
23     (modify ?next:flag TRUE)
24     (alarm :after 10000 :content ())
25   )
26
27   (rule send_task_notification_2
28     (data)
29     (next :process ?proc :flag FALSE) = ?next
30     (search) = ?search
31     (process :flag TRUE)
32     (repository :name ?name)
33     (state :name "F2_1") = ?state
34     -->
35     (modify ?search:action ?next:process)
36     (send
37       :performative "task notification"
```

```
      :to _broadcast
      :arrival ?name
      :content ?search
    )
    (modify ?state:name "F2_1-wait")
    (modify ?next:flag TRUE)
    (alarm :after 10000 :content ())
  )

  (rule send_task_notification_3
    (data :unit ?unit :flag TRUE) = ?data
    (search) = ?search
    (process :flag FALSE)
    (repository :name ?name)
    (state :name "F2_1") = ?state
    -->
    (send
      :performative "task notification"
      :to _broadcast
      :arrival ?name
      :content ?search
    )
    (modify ?state:name "F2_1-wait")
    (remove ?search)
    (alarm :after 10000 :content ())
  )

  (rule send_task_notification_4
    (data :unit ?unit :flag TRUE)
    (next :process ?proc :data ?unit :flag FALSE) = ?next
    (next :process ?proc :flag TRUE)
    (process :flag TRUE)
    (repository :name ?name)
    (state :name "F2_1")
    -->
    (modify ?next:flag TRUE)
  )

  (rule receive_alarm_1
    (Msg :performative __Alarm) = ?alarm
    (state :name "F2_1-wait") = ?state
    -->
    (modify ?state:name "F2_1")
    (remove ?alarm)
  )

  (rule no_task_1
    (data :unit ?unit :flag TRUE) = ?data
    (next :data ?unit :flag TRUE)
    (next :data ?unit :flag FALSE)
    (process :flag TRUE)
    (state :name "F2_1") = ?state
    -->
    (modify ?state:name "F2_2")
  )


  (rule no_task_2
    (data)
    (state :name "F2_1") = ?state
    -->
    (modify ?state:name "F2_2")
  )

  (rule no_task_3
    (data)
    (data :index NONE)
    (process :flag FALSE)
    (state :name "F2_1") = ?state
```

```
    -->
    (modify ?state:name "F2_2")
  )

  (rule no_task_4
    (next)
    (state :name "F2_1") = ?state
    -->
    (modify ?state:name "F2_2")
  )

  (rule send_related_word
    (search :device ?dev :relevance ?rel) = ?req
    (relatedword :wordA ?dev :wordB ?wordB :relevance ?rel2) = ?relword
    (repository :name ?name)
    (state :name "F2_2") = ?state
    -->
    (bind ?nextRel (* ?rel ?rel2))
    (send
      :performative "request notification"
      :to _broadcast
      :arrival ?name
      :content (search :device ?wordB :target ?req:target :action ?req:action
           :relevance ?nextRel :from DSAgent)
    )
    (alarm :after 10000 :content ())
    (remove ?relword)
    (modify ?state:name "F2_2-wait")
  )

  (rule receive_alarm_2
    (Msg :performative __Alarm) = ?alarm
    (state :name "F2_2-wait") = ?state
    -->
    (modify ?state:name "F2_2")
    (remove ?alarm)
  )

  (rule sended_all_related_words_1
    (search :device ?dev)
    (relatedword :wordA ?dev :wordB ?wordB :relevance ?rel2)
    (state :name "F2_2") = ?state
    -->
    (modify ?state:name "F3")
  )

  (rule sended_all_related_words_2
    (search :device ?dev)
    (relatedword :wordA ?dev :wordB ?wordB :relevance ?rel2)
    (state :name "F2_2") = ?state
    -->
    (modify ?state:name "F3")
  )
)
```

1.5. F3: Data Processing Squence Organization

Listing S5 is the rule set for F3: data processing sequence organization. In this rule set, the rule on line 77 is a rule that fires when there is a message addressed to itself with the performative attribute of "`task notification`". When this rule fires, the procedures necessary for cooperating with other agents, described from lines 105 to 169, are executed. If there is no message addressed to itself with a performative attribute of "`task notification`," the rule on line 94 fires and this data processing agent disappears from the operating environment.

Listing S5: Rule set file of F3

```
(rule-set F3Rules

  (property)
  (initial_facts)

  (rule receive_request_acceptance
    (Msg
      :performative "request acceptance"
      :to ?myName
      :content (accepted :by ?agName)
    ) = ?msg
    (Status :name ?myName)
    (notification :from ?notiName :departure ?dep)
    -->
    (make (receiver :name ?agName))
    (send
      :performative "request acceptance"
      :to ?notiName
      :arrival ?dep
      :content ?msg:content
    )
    (remove ?msg)
  )

  (rule receive_file_path_1
    (Msg
      :performative "sending file path"
      :to ?myName
      :content (file :path ?path :by ?agName)
    ) = ?msg
    (Status :name ?myName)
    (receiver :name ?agName) = ?reciever
    (notification :from ?notiName :departure ?dep)
    -->
    (send
      :performative "sending file path"
      :to ?notiName
      :arrival ?dep
      :content ?msg:content
    )
    (remove ?reciever)
    (remove ?msg)
  )

  (rule receive_processing_failure
    (Msg
      :performative "processing failure"
      :to ?myName
      :content (failed :by ?agName)
    ) = ?msg
    (Status :name ?myName)
    (receiver :name ?agName) = ?reciever
    (notification :from ?notiName :departure ?dep)
    -->
    (send
      :performative "processing failure"
      :to ?notiName
      :arrival ?dep
      :content ?msg:content
    )
    (remove ?reciever)
    (remove ?msg)
  )

  (rule receive_all_file
    (Msg
      :performative "request acceptance"
      :to ?myName
```

```
    )
    (receiver :name ?agName)
    (data :index NONE)
    (state :name F3) = ?state
    -->
    (modify ?state:name "processing")
  )

  (rule receive_task_acceptance
    (Msg
      :performative "task acceptance"
      :to ?myName
    ) = ?msg
    (Status :name ?myName)
    (type) = ?type
    -->
    (make (accepter :name ?msg:from :flag FALSE))
    (reply
      :performative "setting information"
      :to ?msg
      :content ?type
    )
    (remove ?msg)
  )

  (rule no_task_acceptance_1
    (Msg
      :performative "task acceptance"
      :to ?myName
    )
    (Status :name ?myName)
    (Msg :performative __Alarm)
    -->
    (alarm :after 3000 :content ())
  )

  (rule receive_setting_information_1
    (Msg
      :performative "setting information"
      :to ?myName
      :content ?cont
    ) = ?msg
    (Status :name ?myName)
    (accepter)
    (state :name "F3") = ?state
    -->
    (print ?cont)
    (control setup(?cont:host ?cont:port ?cont:topic))
    (reply
      :performative "ready"
      :to ?msg
      :content ()
    )
    (modify ?state:name "processing")
    (remove ?msg)
  )

  (rule receive_setting_information_2
    (Msg
      :performative "setting information"
      :to ?myName
      :content ?cont
    ) = ?msg
    (Status :name ?myName)
    (accepter :flag TRUE)
    (accepter :flag FALSE)
    (Msg :performative "request acceptance")
    (state :name "F3") = ?state
    -->
```

```
    (control setup(?cont:host ?cont:port ?cont:topic))
    (reply
      :performative "ready"
      :to ?msg
      :content ()
    )
    (modify ?state:name "processing")
    (remove ?msg)
  )

  (rule receive_ready
    (Msg
      :performative "ready"
      :from ?agName
      :to ?myName
    ) = ?msg
    (Status :name ?myName)
    (accepter :name ?agName :flag FALSE) = ?accepter
    -->
    (modify ?accepter:flag TRUE)
    (remove ?msg)
  )

  (rule receive_all_ready
    (accepter :flag TRUE)
    (accepter :flag FALSE)
    (Msg :performative "request acceptance")
    (data)
    (state :name "F3") = ?state
    -->
    (modify ?state:name "processing")
  )
)
```

1.6. Post processing rules

Listing S6 is the rule set for post processing. Post processing defines the processing to be executed after the DPA processing task is completed. Line 6 is a rule that fires when the agent receives the data processing result from the agent that received the data output by itself. Here, the path to the file containing the processed results is passed to the base process. Line 21 is a rule that fires when processing results are passed from all agents that have received data output by itself. This rule aggregates the processing results and passes them to the other data processing agent that called it or his UI agent. When all post-processing is completed, the data processing agent disappears from the operating environment.

Listing S6: Rule set file of post processing

```
1  (rule-set PostProcessingRules
2
3    (property)
4    (initial_facts)
5
6    (rule receive_file_path_2
7      (Msg
8        :performative "sending file path"
9        :from ?agName
10       :to ?myName
11       :content (file :path ?filePath)
12     ) = ?msg
13     (accepter :name ?agName :flag TRUE) = ?accepter
14     (Status :name ?myName)
15     -->
16     (control receiveFilePath(?filePath))
17     (modify ?accepter:flag END)
18     (remove ?msg)
19   )
20
```

```
  (rule send_result_1
    (accepter :flag END)
    (accepter :flag TRUE)
    (notification)= ?noti
    (file) = ?file
    -->
    (control makeResultFile())
    (send
      :performative "sending file path"
      :to ?noti:from
      :arrival ?noti:departure
      :content ?file
    )
    (remove ?file)
    //(terminate :at now)
  )

  (rule send_result_2
    (receiver)
    (Msg :performative __Alarm :content (request :acceptance END))
    (RWAlarm)
    (data :index NONE)
    (notification) = ?noti
    (file) = ?file
    -->
    (control makeResult())
    (send
      :performative "sending file path"
      :to ?noti:from
      :arrival ?noti:departure
      :content ?file
    )
    //(terminate :at now)
  )

)
```

2. Commonly Used Class Files in Base Process

2.1. Data Proces Module

Listing S7 is a class file to define data process module which is commonly used in the base processes.

Listing S7: Class file for base process

```
public class DataProcessingAgent implements DashBP {
    protected DashAgent dashAgent = null;
    protected Worker worker = null;
    protected ZmqSub input = null;
    protected ZmqPub output = null;

    protected Thread th = null;
    protected List<String> pathList;
    protected String url, path = "";

    @Override
    public void setAgent(DashAgent dashAgent) {
        this.dashAgent = dashAgent;
        output = OutputSetting.outputSetting(dashAgent);
        pathList = new ArrayList<>();
    }

    @Override
    public void finalizeBP() {
        if (worker != null) {
            worker.stop();
            worker.dispose();
        }
```

```
    }

    @Override
    public void run() {
        if (worker != null) {
            worker.start();
        }
    }

    public void start() {
        th = new Thread(this);
        th.start();
    }

    public void stop() {
        finalizeBP();
    }

    public void makePathName(Object[] obj) {
        makePathName(String.valueOf(obj[0]), String.valueOf(obj[1]));
    }

    private void makePathName(String basePath, String num) {
        String resultPath = basePath + num + ".html";
        setPath(resultPath);
        dashAgent.raiseEvent("file :path \"" + resultPath + "\"");
    }

    public void setURL(Object[] obj) {
        setURL(String.valueOf(obj[0]));
    }

    public void setURL(String url) {
        this.url = url;
    }

    public void setPath(Object[] obj) {
        setPath(String.valueOf(obj[0]));
    }

    public void setPath(String path) {
        this.path = path;
    }

    public void setup(Object[] obj) {
        try {
            String address = InetAddress.getByName(String.valueOf(obj[0])).
                getHostAddress();
            int port = Integer.parseInt(String.valueOf(obj[1]));
            String agentName = String.valueOf(obj[2]);
            setup(address, port, agentName);
        } catch (UnknownHostException e) {
            e.printStackTrace();
        }
    }

    private void setup(String address, int port, String agentName) {
        input = new ZmqSub(new ZmqSetting(ZmqProtocols.TCP, address, port,
            false), agentName);
    }

    public void receiveFilePath(Object[] obj) {
        receiveFilePath(String.valueOf(obj[0]));
    }

    private void receiveFilePath(String filePath) {
        pathList.add(filePath);
    }

```

```
    public void judge(Object[] obj) {
        judge(String.valueOf(obj[0]), String.valueOf(obj[1]));
    }

    public void judge(String word1, String word2) {
        Tokenizer tokenizer = new Tokenizer();
        List<Token> tokens = tokenizer.tokenize(word1);
        int counter = 0;
        int match = 0;
        for (Token token : tokens) {
            String surface = token.getSurface();
            String speechLevel = token.getPartOfSpeechLevel1();
            if (speechLevel.equals("noun")) {
                if (word2.contains(surface)) {
                    match++;
                }
                counter++;
            }
        }

        if (match == counter) {
            dashAgent.raiseEvent("TRUE");
        } else {
            double border = (1 - (match / counter)) / 2.0;
            Random rand = new Random();
            double value = rand.nextDouble();
            if (border <= value) {
                dashAgent.raiseEvent("TRUE");
            } else {
                dashAgent.raiseEvent("FALSE");
            }
        }
    }

    public void judgeProcess(Object[] obj) {
        judgeProcess(String.valueOf(obj[0]), Double.valueOf(String.valueOf(
            obj[1])));
    }

    public void judgeProcess(String word, Double score) {
        double border = 1.0 - score;
        Random rand = new Random();
        double value = rand.nextDouble();
        String content = "process :type \"" + word + "\" :flag ";
        if (border <= value) {
            dashAgent.raiseEvent(content + "TRUE");
        } else {
            dashAgent.raiseEvent(content + "FALSE");
        }
    }

    public void makeResultFile() {
        try {
            PrintWriter result_pr = new PrintWriter(new FileWriter(path));
            String content_line;
            for (String url : pathList) {
                BufferedReader content_br = new BufferedReader(new FileReader
                    (url));
                while ((content_line = content_br.readLine()) != null) {
                    result_pr.println(content_line);
                }
                content_br.close();
            }
            pathList.clear();
            result_pr.close();
        } catch (IOException e) {
            e.printStackTrace();
        }
    }
```

```
}
```

2.2. Data Proces Module

Listing S8 is a class file to define data-process module which is commonly used in the base processes. This class file uses the `PostEvent` method, which takes character strings or byte array data as arguments, to pass data from the receiving module to the data processing module and from the data processing module to the sending module.

Listing S8: Data process module

```
public class PostEvent extends EventObject {
    private byte[] data;

    public PostEvent(Object source, String message) {
        super(source);
        this.data = message.getBytes(Charset.forName("UTF-8"));
    }

    public PostEvent(Object source, byte[] data) {
        super(source);
        this.data = data;
    }

    public String getMessage(){
        return new String(data,Charset.forName("UTF-8"));
    }

    public byte[] getData(){
        return data;
    }
}
```

Reference

1. Uchiya, T.; Kinoshita, T. Design support functions for developing multiagent system on repository-based multiagent framework. *2011 10th IEEE International Conference on Cognitive Informatics & Cognitive Computing (ICCI*CC)* **2011**, 240–246.
